# Commensal yeast *Malassezia* produces tryptophan metabolites to promote tissue homeostasis via the aryl hydrocarbon receptor in mice

**DOI:** 10.1038/s41564-026-02382-8

**Published:** 2026-06-19

**Authors:** Eduardo Gushiken-Ibañez, Michelle Stokmaier, Giuseppe Barone, Alessia Staropoli, Tugay Karakaya, Hans-Dietmar Beer, Francesco Vinale, Giuseppe Ianiri, Salomé LeibundGut-Landmann

**Affiliations:** 1 Section of Immunology, Vetsuisse Faculty, University of Zurich, Zurich, Switzerland; 2 Institute of Experimental Immunology, University of Zurich, Zurich, Switzerland; 3 Department of Agricultural, Environmental and Food Sciences, Università degli Studi del Molise, Campobasso, Italy; 4 Department of Veterinary Medicine and Animal Productions, University of Naples Federico II, Naples, Italy; 5 Department of Dermatology, University Hospital Zurich, Schlieren, Switzerland; 6 Medical Research Council Centre for Medical Mycology, Department of Biosciences, Faculty of Health and Life Sciences, University of Exeter, Exeter, UK

**Keywords:** Fungal host response, Fungal biology

## Abstract

As an abundant fungal colonizer of mammalian skin, *Malassezia* establishes mutualistic or pathogenic interactions with the host. Here we show that *Malassezia furfur* promotes skin homeostasis by maintaining epidermal integrity via tryptophan-derived metabolites that activate the aryl hydrocarbon receptor (AhR), a key regulator of keratinocyte differentiation and inflammation. *M. furfur*-derived tryptophan derivatives activated AhR in human epidermal equivalents and upregulated proteins important for skin structure and barrier activity in mouse epidermis. In a mouse model of atopic dermatitis, *M. furfur* colonization with tryptophan supplementation reduced inflammation and restored barrier function, while a fungal mutant defective in indole production was unable to do so. Mice lacking AhR specifically in keratinocytes failed to benefit from *M. furfur*-mediated barrier protection. These findings establish a previously unrecognized mutualistic role for *Malassezia* in skin physiology and expand our understanding of the skin microbiota’s influence on barrier function and immune regulation.

## Abstract (web summary)

A ubiquitous skin fungus, frequently associated with skin pathology, is important for maintenance of the skin barrier function through tryptophan derivatives that act as aryl hydrocarbon receptor agonists.

## Main

The skin forms a critical barrier against environmental threats, with the epidermis having a central role in limiting water loss and preventing harmful exposure^1^. Disruption of this barrier is a hallmark of common skin disorders such as atopic dermatitis and psoriasis^2,3^, highlighting the importance of mechanisms that maintain and restore skin barrier integrity. However, the relative contributions of host-intrinsic factors and microbial influences to these processes remain incompletely understood. The aryl hydrocarbon receptor (AhR) is a key regulator of skin homeostasis, integrating environmental cues to detoxify xenobiotic compounds, modulate immune defences and regulate keratinocyte differentiation and function^4^. Following activation, the AhR translocates to the nucleus and drives transcriptional programmes promoting the skin barrier function^5,6^.

Members of the microbiota have emerged as a source of AhR ligands, including *Staphylococcus* spp.^7^, *Corynebacterium* spp.^8^ and the yeast *Malassezia*^9,10^. *Malassezia* spp. dominate the human skin mycobiome and are highly adapted to lipid-rich niches^11^. Of the 21 species cultivated to date, *Malassezia restricta* and *Malassezia globosa* are most abundant on human skin, along with other species such as *Malassezia arunalokei*, *Malassezia sympodialis* and *Malassezia furfur*^12^. Despite *Malassezia*’s commensal lifestyle, some species including *M. furfur* are associated with common inflammatory skin disorders, such as atopic dermatitis, seborrhoeic dermatitis and pityriasis versicolor^13^, and rare septic infection^14^, although their role in disease pathogenesis remains unclear, with evidence suggesting both detrimental and protective effects^15–18^.

*Malassezia* metabolizes tryptophan, which is naturally present in the skin as a component of sweat^19^, into indolic compounds that activate the AhR^9,10,20^, yet the functional relevance of these fungal metabolites in skin physiology is not well defined. Given the abundance of *Malassezia* on human skin, the indoles produced may have an important role in shaping skin physiological processes.

In this Article, we show that *Malassezia*-derived indoles activate AhR signalling in keratinocytes, promoting barrier restoration in inflamed and disrupted skin. This effect is dependent on keratinocyte-intrinsic AhR activation and is lost when using *Malassezia*mutant strains deficient in the production of AhR-activating indoles, but can be rescued by exogenous indoles. These findings identify *Malassezia* as a contributor to skin homeostasis through the production of AhR agonists, highlighting a beneficial role for this commensal fungus.

## Results

### *M. furfur*-derived metabolites induce strong AhR activation

**Fig. 1:**
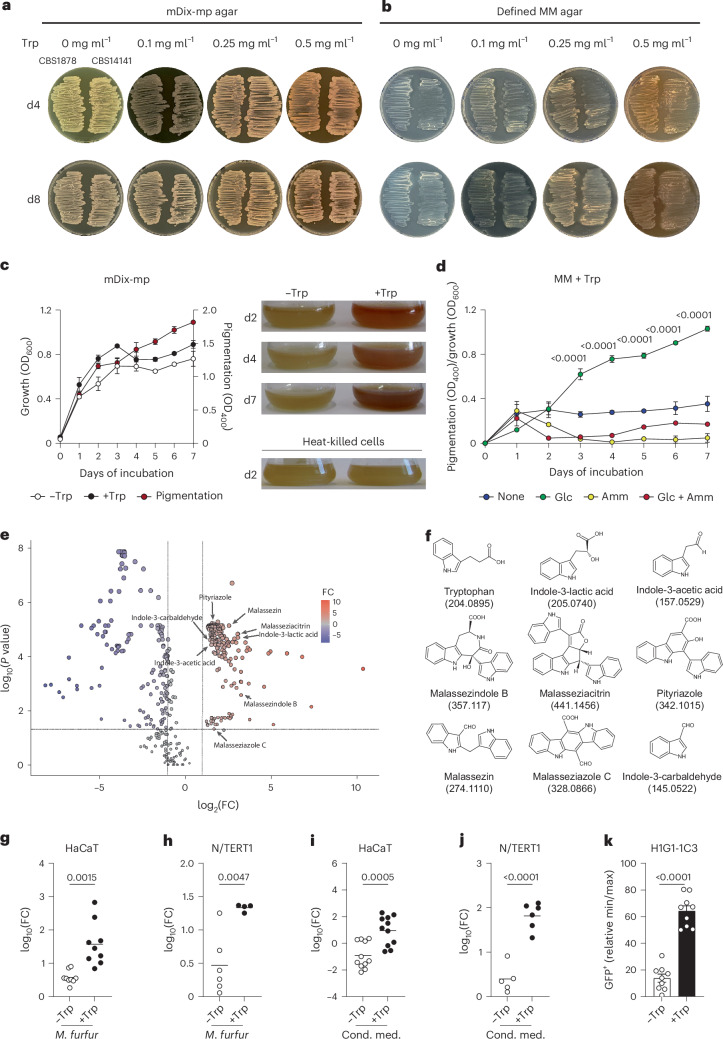
*M. furfur*-derived metabolites induce strong AhR activation via production of indoles in a tryptophan-dependent manner. **a**,**b**, *M. furfur* strains CBS1878 and CBS14141 grown for 4 days and 8 days on mDix-mp agar (**a**) or MM agar (**b**) supplemented with 0 mg ml^−1^, 0.1 mg ml^−1^, 0.25 mg ml^−1^ and 0.5 mg ml^−1^ of L-tryptophan. **c**, *M. furfur* CBS14141 growth (OD_600_, left axis) and pigment release (OD_400_, right axis) at the indicated days (d) of incubation in liquid mDix-mp in the presence or absence of L-tryptophan. *n* = 3 biological replicates per group. The mean ± s.e.m. is indicated. **d**, *M. furfur* CBS14141 pigment production relative to the growth (OD_400_/OD_600_) in liquid MM supplemented with tryptophan and glucose as carbon source (Glc), ammonium nitrate as nitrogen source (Amm), glucose and ammonium nitrate (Glc + Amm) or tryptophan as sole carbon and nitrogen source (Trp). *n* = 3 biological replicates per group. The mean ± s.e.m. is indicated. The statistical significance of differences between groups was determined by two-way ANOVA. The highest *P* value among the comparisons between the glucose group and the other groups is indicated. **e**,**f**, Untargeted metabolomics of *M. furfur* CBS14141 grown for 4 days in mDix-mp supplemented with or without tryptophan. A volcano plot of metabolites differentially produced by *M. furfur* when grown in the presence of tryptophan (**e**) and the structure of detected indoles produced by *M. furfur* (the molecular weight is indicated in brackets, **f**) are shown. *n* = 6 per group. The statistical significance of differences between groups was determined by unpaired two-tailed *t-*test with FDR correction (cut-off = 0.05) and by FC ± 2.0. **g**–**j**, *CYP1A1* expression by HaCaT (**g**,**i**) and N/TERT1 (**h**,**j**) keratinocyte cell lines after 24 h of infection with *M. furfur* CBS14141 (**g**,**h**) or exposure to conditioned medium(Cond. med.) of *M. furfur* CBS14141 (**i**,**j**) grown in the presence (+) or absence (−) of tryptophan. Data are plotted as the fold change over unstimulated controls. Each dot represents one biological replicate; **g**: *n* = 9 per group pooled from 3 independent experiments; **h**: *n* = 6 (−Trp) or *n* = 4 (+Trp); **i**: *n* = 11 per group pooled from 5 independent experiments; **j**: *n* = 5 (−Trp) or *n* = 6 (+Trp) pooled from 2 independent experiments. The mean of each group is indicated. The statistical significance of differences between groups was determined by unpaired two-tailed *t-*test. **k**, GFP expression by the reporter cell line H1G1-1C3 in response to 24 h of stimulation with conditioned medium from *M. furfur* CBS14141 (**i**,**j**) grown in the presence (+) or absence (−) of tryptophan. Expression levels are shown relative to unstimulated (min) and 6-formylindolo(3,2-b)carbazole (FICZ, max)-stimulated cells. Each symbol represents one independently stimulated well; bars indicate the mean. *n* = 9 per group, pooled from 2 independent experiments. The statistical significance of differences between groups was determined by unpaired two-tailed *t*-test. See also Extended Data Fig. 1.

**Extended Data Fig. 1:**
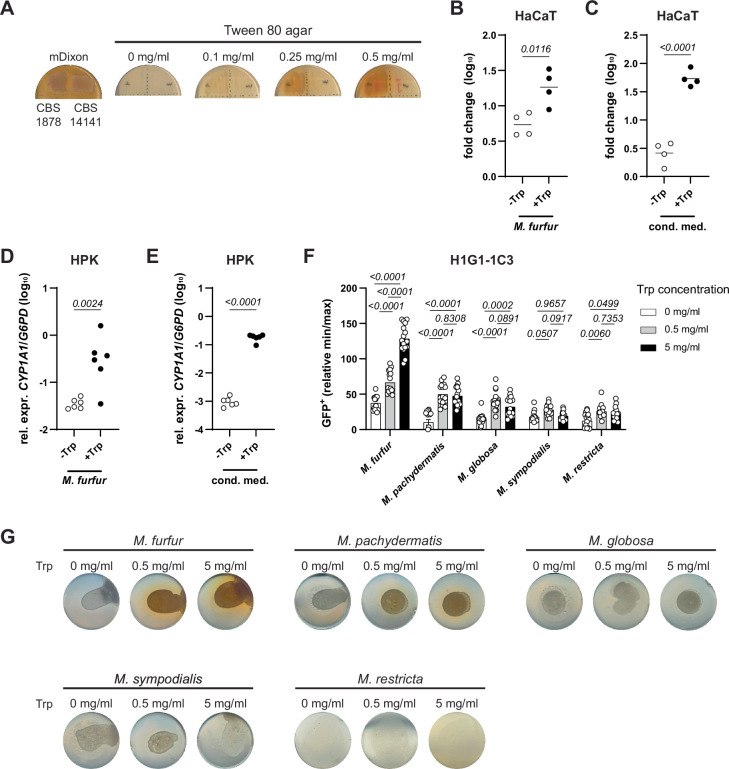
*M. furfur*-derived metabolites induces strong AhR activation via production of indoles in a tryptophan dependent manner. **A**. *M. furfur* strains CBS1871 and CB14141 grown for 8 days on Tween 80 agar supplemented with 0, 0.1, 0.25 and 0.5 mg/mL L-tryptophan. **B. – C**. *CYP1A1* expression by HaCaT keratinocyte cell lines after 6 hours of infection with *M. furfur (B) or exposure to conditioned medium of M. furfur* CB14141 *(C)* grown in presence ( + Trp) or absence (-Trp) of tryptophan. Data are plotted as the fold change over the mean of unstimulated controls. n = 4 / group. The mean of each group is indicated. The statistical significance of differences between groups was determined by unpaired two-tailed *t-*test (B, C). **D. – E**. *CYP1A1* expression by human primary keratinocytes (HPK) after 24 hours of infection with *M. furfur cells (D) or conditioned medium from M. furfur* CB14141 *(E)*, grown in presence (+) or absence (-) of tryptophan. n = 6 / group pooled from 2 independent experiments. The mean of each group is indicated. The statistical significance of differences between groups was determined by unpaired two-tailed *t*-test. **F**. GFP expression by the reporter cell line H1G1-1C3 in response to 24 hours of stimulation with *conditioned medium from the indicated Malassezia spp*. grown without tryptophan or in presence of 0.5 or 5 mg/ml tryptophan. Expression levels are displayed relative to unstimulated and FICZ-stimulated cells. Each symbol represents one independently stimulated well; bars indicate the mean. n = 16 pooled from 2 independent experiments.The statistical significance of differences between groups was determined by two-way ANOVA. **G**. Representative images of *Malassezia* spp. grown on Tween 80 agar plates supplemented with 0, 0.5 and 5 mg/ml L-tryptophan.

To assess the ability of *Malassezia* to metabolize tryptophan, we evaluated two different strains of *M. furfur*—a species known for its strong indole-producing capacity—under three distinct growth conditions. On Tween 80 agar, only *M. furfur* CBS1878 showed strong production of brown pigments, corroborating previous findings^20^ (Extended Data Fig. 1a). However, both strains CBS1878 and CBS14141 produced pigments in an L-tryptophan dose-dependent manner in modified Dixon medium without mycological peptone (mDix-mp, reported in ref. ^21^) and in a defined minimal medium (MM) (Fig. 1a,b). Pigments were observed both intracellularly, resulting in brown-coloured cells, and secreted into the medium, suggesting an active process of pigment production and release.

Further experiments were carried out using only the haploid *M.* *furfur* strain CBS14141, which has a high-quality genome assembly^22^ and is amenable to genetic manipulation^23,24^. Growth (measured as optical density at 600 nm, OD_600_) and pigment production (measured as optical density at 400 nm, OD_400_) were quantified in liquid mDix-mp supplemented with tryptophan. Pigment production increased over time from day 2 to day 7 (Fig. 1c), in contrast to cultures grown without tryptophan, which remained pale. Heat-killed cells were unable to produce pigments, indicating that tryptophan degradation depended on metabolic activity (Fig. 1c). Pigment production was highest when tryptophan served as a nitrogen source in the presence of glucose or Tween 80 as an additional carbon source (Fig. 1d and Extended Data Fig. 1a), indicating the influence of nutrient availability and metabolic context on indole biosynthesis, consistent with previous results^20^.

Untargeted metabolomics of *M. furfur* CBS14141 revealed a substantial shift in the metabolic profile following tryptophan supplementation, with 256 upregulated and 83 downregulated compounds (false discovery rate (FDR) < 0.05; log_2_(fold change (FC) ± 2); Fig. 1e and Supplementary Table 1). Among these, eight were identified exclusively when *M. furfur* was grown in the presence of tryptophan, including the previously described *Malassezia*-derived AhR ligands indole-3-lactic acid, malasseziazole C, malassezindole B, indole-3-carbaldehyde, pityriazole, malassezin^25^, indole-3-acetic acid and malasseziacitrin^26^ (Fig. 1f).

To determine whether these metabolites function as AhR agonists, we exposed the human keratinocyte cell lines HaCaT and N/TERT1 to *M. furfur* CBS14141 grown in the presence or absence of tryptophan and assessed AhR activation via expression of *CYP1A1*. *CYP1A1* expression was strongly enhanced when keratinocytes were stimulated for 6 h with fungal cells or with conditioned medium prepared from *M. furfur* grown in the presence of tryptophan, confirming that the effect was due to molecules contained within the secretome of *M. furfur* (Extended Data Fig. 1b,c). The response remained detectable at 24 h (Fig. 1g–j). Induction of *CYP1A1* expression by *M. furfur*-derived metabolites was also conserved in primary human keratinocytes (Extended Data Fig. 1d,e) and further confirmed by means of an AhR reporter cell line (H1G1-1C3 (ref. ^27^)) (Fig. 1k). The capacity to activate AhR signalling through tryptophan-dependent metabolites is not unique to *M. furfur*, which, however, showed stronger AhR activation compared with other *Malassezia* species tested, including *Malassezia pachydermatis*, *M. globosa*, *M. restricta* and *M. sympodialis*, regardless of the tryptophan concentration used (Extended Data Fig. 1f,g). Together, these findings corroborate that *M. furfur* possesses an enhanced capacity to metabolize tryptophan and that *M. furfur*-derived tryptophan metabolites are potent AhR agonists, directly binding to AhR and activating downstream signalling.

### Activation of AhR by *M. furfur* in the epidermis modulates the expression of structural protein-encoding genes

**Fig. 2:**
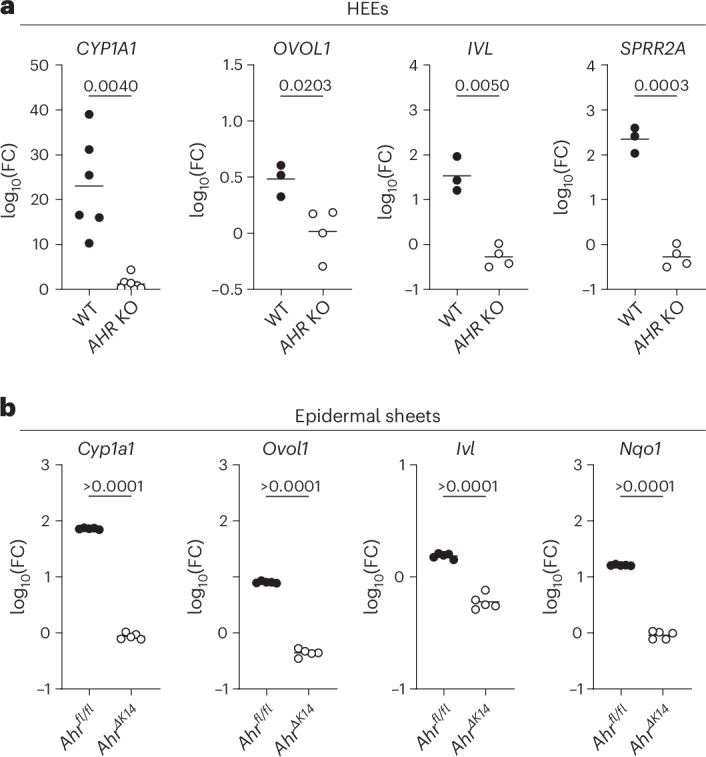
Activation of *AhR* by *M. furfur* in the epidermis modulates the expression of structural protein-encoding genes. **a**, *CYP1A1*, *OVOL1*, *IVL* and *SPRR2A* expression by HEEs generated from WT or AhR-deficient N/TERT1 cells after 24 h of stimulation with conditioned medium from *M. furfur* CBS14141 grown with tryptophan. Data are plotted as the fold change over the mean of the unstimulated controls. *n* = 6 (WT) or *n* = 8 (KO) pooled from 2 independent experiments for *CYP1A1* and *n* = 3 (WT) or *n* = 4 (KO) for *OVOL1*, *IVL* and *SPRR2A*. The mean of each group is indicated. The statistical significance of differences between groups was determined by unpaired two-tailed *t-*test. **b**, *Cyp1a1*, *Ovol1*, *Ivl* and *Nqo1* expression by epidermal sheets obtained from the ear skin of *Ahr*^*fl/fl*^ or *Ahr*^Δ*K14*^ mice after 24 h of incubation in conditioned medium from *M. furfur* CBS14141 grown with tryptophan. Data are plotted as the FC over the mean of the unstimulated controls. *n* = 5 per group pooled from 2 independent experiments. The mean of each group is indicated. The statistical significance of differences between groups was determined by unpaired two-tailed *t-*test. See also Extended Data Fig. 2.

**Extended Data Fig. 2:**
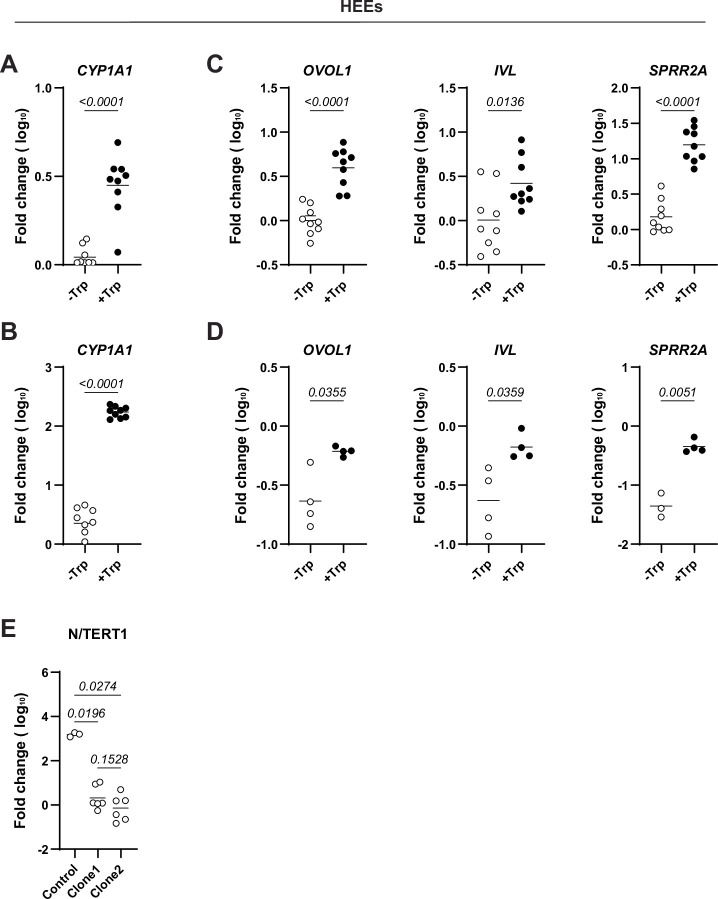
Activation of *AhR* by *M. furfur* in the epidermis modulates the expression of structural protein encoding genes. **A. - D**. *CYP1A1* (A, B) and *OVOL1*, *IVL*, and *SPRR2A* expression (C, D) by WT HEEs after 24 hours of infection with *M. furfur* CB14141 *(A, C) or exposure to conditioned medium from M. furfur* CB14141 *(B, D)* grown with or without tryptophan. Data are plotted as the fold change over the mean of unstimulated controls. For A – C, n = 9 / group pooled from two independent experiments; for D, n = 4 / group. The mean of each group is indicated. The statistical significance of differences between groups was determined by unpaired two-tailed *t-*test. **E**. *CYP1A1* expression by two independent clones of *AHR* KO N/TERT1 and by parental WT N/TERT1 keratinocytes after 24 hours of stimulation with FICZ. Data are plotted as the fold change over unstimulated controls. n = 3 / group for WT, n = 6 / group for AHR KO clone 1 and clone 2 / group. The mean of each group is indicated. The statistical significance of differences between groups was determined by ne-way ANOVA.

We next examined the functional consequences of *Malassezia*-induced AhR activation in the skin. Because keratinocyte monolayer cultures do not reproduce the stratified epidermis well, we generated human epidermal equivalents (HEEs) using air–liquid interface cultures of N/TERT keratinocytes^28^. Consistent with data from keratinocyte monolayers, both tryptophan-processing fungal cells and their soluble metabolites strongly induced AhR signalling in HEEs, as evidenced by increased *CYP1A1* expression (Extended Data Fig. 2a,b). Importantly, we also found upregulation of genes critical for barrier function, including involucrin (*IVL*), small proline-rich protein 2A (*SPRR2A*) and the transcriptional regulator of keratinocyte differentiation *OVOL1* (ref.^29^; Extended Data Fig. 2c,d), consistent with a role of AhR in enhancing keratinocyte differentiation and barrier integrity. To confirm AhR dependence, *AHR* was mutated in N/TERT1 cells using clustered regularly interspaced short palindromic repeats (CRISPR)–CRISPR-associated protein 9 (Cas9) gene editing (Extended Data Fig. 2e), and HEEs were generated from knockout cells. As expected, *AHR*-deficient HEEs failed to induce *CYP1A1*, *OVOL1*, *IVL* and *SPRR2A* expression in response to tryptophan-dependent fungal metabolites (Fig. 2a).

These findings were validated in epidermal sheets from mouse ear skin, which retain native tissue architecture, including immune cells. Stimulation with tryptophan-dependent fungal metabolites triggered the induction of *Ovol1*, *Ivl* and *Nqo1*, which reflects Nrf2 pathway activation^30^ (Fig. 2b). This response was abolished in epidermal sheets from *Ahr*^*ΔK14*^ mice lacking AhR selectively in keratinocytes, showing that AhR signalling was keratinocyte intrinsic (Fig. 2b). Together, these results suggested that *M. furfur*-induced tryptophan-dependent metabolites have the capacity to modulate skin barrier architecture through AhR-dependent signalling in keratinocytes.

### *M. furfur*-induced AhR signalling restores barrier integrity of inflamed skin

**Fig. 3:**
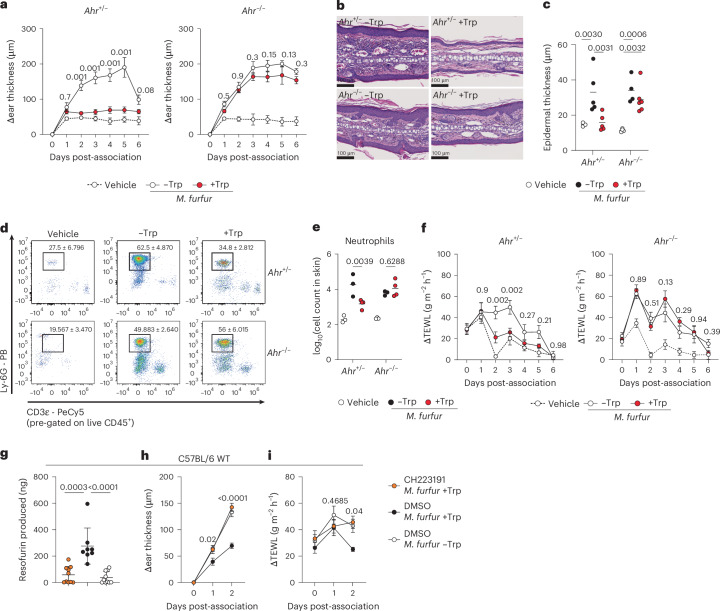
*M. furfur-*induced AhR signalling restores barrier integrity in inflamed skin. **a**–**i**, The ear skin of *Ahr*^−/−^ mice and *Ahr*^*+/*−^ littermate control mice (**a**–**f**) or of WT C57BL/6 mice (**g**–**i**) was mildly tape stripped before epicutaneous administration of *M. furfur* CBS14141 grown in the presence (+Trp) or absence of tryptophan (−Trp). A group of control mice was tape stripped and treated with olive oil (vehicle). **a**,**b**, Increase in skin thickness in *Ahr*^−/−^ (**b**) and *Ahr*^*+/*−^ littermate control mice (**a**) at the indicated time point relative to pretreatment. Vehicle: *n* = 4 per group; −Trp: *n* = 8 per group; +Trp: *n* = 9 (*Ahr*^+/−^) or *n* = 10 (*Ahr*^−/−^) pooled from 2 independent experiments. The mean ± s.e.m. is indicated. The statistical significance of differences between the *M. furfur* +Trp and *M. furfur* −Trp groups was determined by two-way ANOVA. **b**,**c**, Representative images of haematoxylin and eosin (H&E)-stained histology sections (**b**) and quantification of epidermal thickness (**c**) on day 7 after fungal association. Vehicle: *n* = 4 per group; −Trp: *n* = 5 (*Ahr*^+/−^) or *n* = 4 (*Ahr*^−/−^); +Trp: *n* = 5 (*Ahr*^+/−^) or *n* = 6 (*Ahr*^−/−^). Each symbol represents the average epidermal thickness per mouse. The mean of each group is indicated. The statistical significance of differences between groups was determined by two-way ANOVA. **d**,**e**, Neutrophils in the ear skin of colonized mice were quantified by flow cytometry 3 days after fungal association. Representative plots (**d**) and neutrophil counts per ear (**e**) are shown. Vehicle: *n* = 3 per group; −Trp: *n* = 3 per group; +Trp: *n* = 4 per group. Each symbol represents one mouse. The statistical significance of differences between groups was determined by two-way ANOVA. **f**, TEWL in *Ahr*^−/−^ (**f**) and *Ahr*^*+/*−^ littermate control mice (**e**) at the indicated time points. Vehicle: *n* = 6 (*Ahr*^+/−^) or *n* = 4 (*Ahr*^−/−^); −Trp: *n* = 8 (*Ahr*^+/^ and *Ahr*^−/−^); +Trp: *n* = 9 (*Ahr*^+/−^) or *n* = 10 (*Ahr*^−/−^) pooled from 2 independent experiments, except for the *Ahr*^−*/*−^ −Trp group. The mean ± s.e.m. is indicated. The statistical significance of differences between the *M. furfur* +Trp and *M. furfur* −Trp groups was determined by two-way ANOVA. **g**–**i**, Mice were treated with CH223191 or DMSO solvent before tape stripping and fungal association. Cyp1a1 activity on day 2 (**g**), increase in skin thickness relative to pretreatment (**h**) and TEWL (**i**) at the indicated time points. *n* = 10 per group pooled from 2 independent experiments. Each symbol represents one mouse (**g**) or the mean ± s.e.m. (**h**,**I**). The statistical significance of differences between groups was determined by one-way ANOVA (**g**) or two-way ANOVA (**h**,**i**). The highest *P* value among the comparisons between the CH223191 group and the other groups is indicated. See also Extended Data Fig. 3.

**Extended Data Fig. 3:**
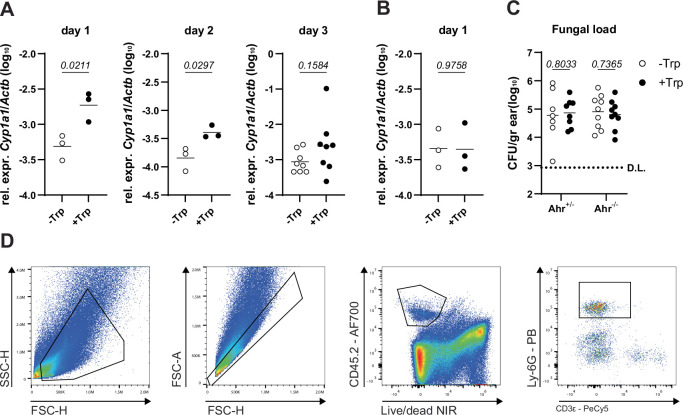
*M. furfur* induced AhR signaling restores barrier integrity in inflamed skin. **A**. The ear skin of C57BL/6 WT mice was mildly tape stripped prior to epicutaneously administering *M. furfur* CB14141, which was pre-cultured in presence (+Trp) or absence of tryptophan (-Trp). *Cyp1a1* relative expression was quantified from the epidermis of the colonized skin at day 1, 2 and 3 after fungal association. n = 3 / group (day 1 and day 2) or n = 8 / group pooled from 2 independent experiments (day 3). The statistical significance of differences between groups was determined by unpaired two-tailed t test. **B**. The ear skin of *Ahr*^−/−^ mice was mildly tape stripped prior to epicutaneously administering *M. furfur* CB14141, which was pre-cultured in presence ( + Trp) or absence of tryptophan (-Trp). *Cyp1a1* relative expression was quantified from the epidermis of the colonized skin at day 1 after fungal association. n = 3 / group. The statistical significance of differences between groups was determined by unpaired two-tailed t test. **C**. The ear skin of *Ahr*^−/−^ and *Ahr*^+/-^littermates control mice was mildly tape stripped prior to epicutaneously administering *M. furfur* CBS14141, which was pre-cultured in presence (+Trp) or absence of tryptophan (-Trp). Fungal load (CFU) was assessed on day 7 after fungal association. N values per group are: -Trp *Ahr*^+/−^ n=7 *Ahr*^−/−^ n=9, +Trp *Ahr*^+/-^ n= 8 *Ahr*^−/−^ n = 9 pooled from 2 independent experiments. The mean of each group is indicated. The statistical significance of differences between groups was determined by two-way ANOVA. **D**. Gating strategy for ear skin neutrophils quantified by flow cytometry in Fig. 3d-e.

On the basis of our in vitro data, we wondered whether *M. furfur* would modulate skin barrier function in vivo. We further speculated that such an effect would be particularly relevant in the context of pathological skin conditions characterized by barrier defects, such as atopic dermatitis^31^. We therefore applied mild tape stripping before associating the ear skin with *M. furfur*^32^. Indole-producing *M. furfur* (+Trp) activated AhR signalling in wild-type (WT) barrier-disrupted mouse skin but not in *Ahr*^−/−^ mice (Extended Data Fig. 3a,b), without affecting fungal colonization (Extended Data Fig. 3c). As shown previously^32^, colonization of barrier-impaired mouse skin with *M. furfur* exacerbated inflammation, reflected by increased skin thickness (Fig. 3a). By contrast, association with indole-producing *M. furfur* suppressed the induction of ear thickness (Fig. 3a), epidermal thickness (Fig. 3b,c) and neutrophil infiltration (Fig. 3d,e and Extended Data Fig. 3d). Importantly, this anti-inflammatory effect was abolished in AhR-deficient mice (Fig. 3a–e).

Barrier disruption leads to increased trans-epidermal water loss (TEWL)^3^. We indeed found a TEWL increase in mice colonized with *M. furfur* grown without tryptophan (Fig. 3f), confirming that the fungus exacerbated the skin barrier defect when not producing indoles. By contrast, indole-producing *M. furfur* limited the TEWL increase (Fig. 3f), supporting a role of *Malassezia*-derived tryptophan metabolites in barrier restoration. This effect was absent in *Ahr*-deficient mice, in which TEWL remained elevated regardless of fungal indole production; baseline changes of TEWL induced by tape stripping alone were AhR independent (Fig. 3f).

To exclude that the observed AhR-dependent phenotype resulted from pre-existing structural and/or functional perturbances in the epidermis due to environmental or dietary AhR ligands^33,34^, we pharmacologically inhibited AhR signalling^35^. In WT C57BL/6 mice, CH223191 treatment effectively blocked AhR activity (Fig. 3g) and abolished the effects of fungal tryptophan metabolites on skin thickness and TEWL (Fig. 3h,i), confirming direct AhR dependence of the *M. furfur*-mediated phenotype.

Together, these results underscore the pivotal role of *Malassezia*-derived metabolites in mediating a host-protective effect during microbial colonization.

### AhR-mediated skin barrier restoration in response to *M. furfur* is keratinocyte intrinsic

**Fig. 4:**
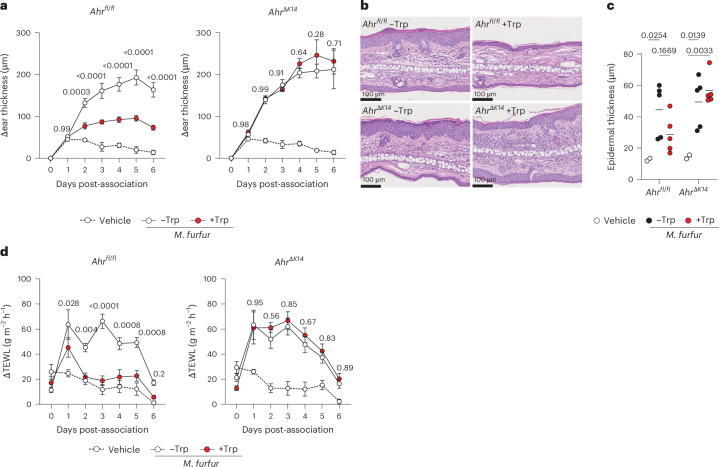
AhR-mediated skin barrier restoration in response to *M. furfur* is keratinocyte intrinsic. The ear skin of *Ahr*^*ΔK14*^ mice and *Ahr*^*fl/fl*^ littermate control mice was mildly tape stripped before epicutaneous administration of *M. furfur* CBS14141 grown in the presence (+Trp) or absence of tryptophan (−Trp). A group of control mice was tape stripped and treated with olive oil (vehicle). **a**, Increase in skin thickness in *Ahr*^*ΔK14*^ and *Ahr*^*fl/fl*^ littermate control mice at the indicated time point relative to pretreatment. *n* = 8 per group pooled from 2 independent experiments, except for the vehicle group in which *n* = 4 (*Ahr*^*fl/fl*^) or *n* = 5 (*Ahr*^*ΔK14*^). The mean ± s.e.m. is indicated. The statistical significance of differences between the *M. furfur* +Trp and *M. furfur* −Trp groups was determined by two-way ANOVA. **b**,**c**, Representative images of H&E-stained histology sections (**b**) and quantification of epidermal thickness (**c**) on day 7 after fungal association. Each symbol represents the average epidermal thickness per mouse. The mean of each group is indicated. *n* = 5 per group pooled from 2 independent experiments, except for the vehicle group in which *n* = 2. The statistical significance of differences between genotypes was determined by two-way ANOVA. **d**, TEWL in *Ahr*^*ΔK14*^ mice and *Ahr*^*fl/fl*^ littermate control mice at the indicated time points. *n* = 8 per group pooled from 2 independent experiments, except for the vehicle group in which *n* = 4 (*Ahr*^*fl/fl*^) or *n* = 5 (*Ahr*^*ΔK14*^). The mean ± s.e.m. is indicated. The statistical significance of differences between the *M. furfur* +Trp and *M*. furfur −Trp groups was determined by two-way ANOVA. See also Extended Data Fig. 4.

**Extended Data Fig. 4:**
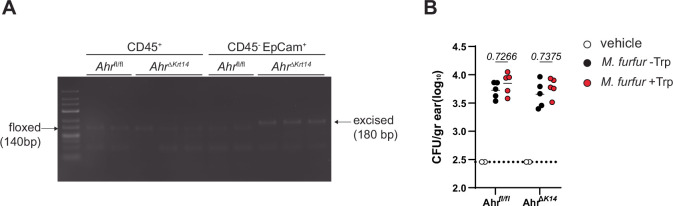
AhR-mediated skin barrier restoration in response to *M. furfur* is keratinocyte intrinsic. **A**. AhR deletion in CD45^+^ and CD45- EpCam^+^ populations sorted from epidermal sheets of naïve *Ahr*^*ΔK14*^ and *Ahr*^*fl/fl*^ littermate control mice. **B**. The ear skin of *Ahr*^*ΔK14*^ mice and *Ahr*^*fl/fl*^ littermate control mice was mildly tape stripped prior to epicutaneously administering *M. furfur* CB14141, which was pre-cultured in presence (+Trp) or absence of tryptophan (-Trp). A group of control mice was tape stripped and treated with olive oil (vehicle). Fungal load (CFU) was assessed on day 7 after fungal association. n = 8 / group pooled from 2 independent experiments, except for the vehicle group where n = 2. The mean of each group is indicated. The statistical significance of differences between groups was determined by two-way ANOVA.

AhR signalling is expressed across various cell subsets in the skin, including keratinocytes, fibroblasts and immune cells. Our in vitro experiment data showing transcriptional changes of barrier genes in keratinocytes (Fig. 2) suggested that keratinocytes may be primary targets of fungal AhR agonists. To confirm that the observed phenotype in vivo occurred keratinocyte intrinsically, we repeated the skin colonization experiments in *Ahr*^*ΔK14*^ mice lacking AhR selectively in keratinocytes, while other cell types retained an intact *Ahr* gene (Extended Data Fig. 4a). Fungal colonization of *Ahr*^*ΔK14*^ mice largely phenocopied what we observed in full-body AhR knockout mice (Fig. 4 and Extended Data Fig. 4b). More specifically, the conditional knockout mice showed elevated inflammation (Fig. 4a), epidermal thickness (Fig. 4b,c) and TEWL (Fig. 4d) irrespective of fungal indole production, whereas cre-negative littermates showed reduced inflammation and improved barrier integrity (Fig. 4a–d).

Together, these findings show that keratinocyte-intrinsic AhR signalling induced by indole-producing *M. furfur* is essential for maintaining skin barrier integrity.

### A forward genetic screen identifies an *M. furfur sul1* mutant with impaired indole production

**Fig. 5:**
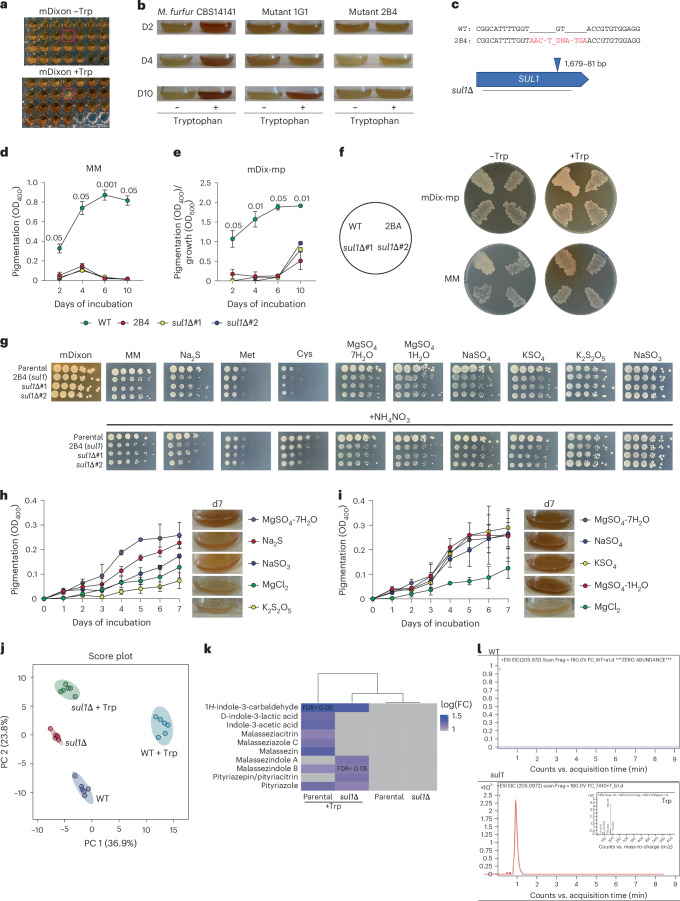
A forward genetic screen identifies an *M. furfur sul1* mutant with impaired indole production. **a**–**c**, Random insertional and targeted mutagenesis in *M. furfur* CBS14141. **a**, Representative insert of a 96-well plate during the screening of random insertional mutants grown in mDix-mp supplemented without (top) or with (bottom) tryptophan; the red square indicates mutant 2B4. **b**, *M. furfur* CBS14141 and two random insertional mutants, strains 1G1 and 2B4, grown in mDix-mp supplemented with or without tryptophan for 2 days, 4 days and 10 days. **c**, Schematic representation of *SUL1* mutagenesis. Top: T-DNA insertion site in the *SUL1* gene in strain 2B4 aligned with the WT sequence. T-DNA sequence in red, including the classical TGA integration sequence of the right border; the inverted triangle represents the position of the T-DNA insertion in the *SUL1* gene, with distance from the start codon indicated in bp. The bar below the gene indicates the region subjected to targeted gene replacement in the *sul1*Δ deletion mutants. **d**–**f**, Pigmentation (OD_400_) and pigmentation relative to the growth (OD_400_/OD_600_) of *M. furfur* CBS14141, *sul1* random insertional mutant (2B4) and targeted mutant (*sul1*Δ#1 and *sul1*Δ#2) in liquid MM with glucose (**d**) and mDix-mp (**e**) supplemented with tryptophan. The same strains were also tested in mDix-mp and MM agar supplemented with or without tryptophan (**f**); the scheme indicates the position of the strains on the plates. In **d** and **e**, *n* = 3. The mean ± s.d. is indicated. The statistical significance of differences between the parental *M. furfur* (WT) and the *sul1* mutants (2B4, *sul1*Δ#1 and *sul1*Δ#2) was determined by two-way ANOVA. The highest *P* value among the comparisons between the WT group and the other groups is indicated. **g**, Growth of *M. furfur* CBS14141 and the 2B4 and *sul1*Δ#1 and *sul1*Δ#2 mutants on MM agar in which MgSO_4_ was replaced with MgCl_2_, and supplemented with 2 mM of the indicated inorganic and organic sulfur sources. **h**,**i**, Pigmentation (OD_400_) of parental *M. furfur* CBS14141 in liquid MM supplemented with MgSO_4_-7H_2_O, Na_2_S, NaSO_3_, MgCl_2_ or K_2_S_2_O_5_ (**h**) or with MgSO_4_-7H_2_O, NaSO_4_, KSO_4_, MgSO_4_.1H_2_O or MgCl_2_ (**i**). *n* = 3. The mean ± s.d. is indicated. **j**–**l**, Untargeted metabolomics of parental *M. furfur* (WT) and *sul1*Δ#1 grown for 4 days in mDixon-mp supplemented with (+Trp) or without tryptophan. **j**, Principal component analysis (PCA) plot of metabolomic data. **k**, Heat map showing the abundance of *M. furfur*-produced indoles. **l**, Extracted ion chromatogram (EIC) relative to the tryptophan signal in parental *M. furfur* (WT) and *sul1*Δ#1 mutant grown in the presence of tryptophan. *n* = 6. In **k**, the statistical significance of differences between WT and *sul1*Δ grown in the presence of tryptophan was determined by unpaired two-tailed *t-*test with FDR correction (cut-off = 0.05). See also Extended Data Fig. 5.

To identify *M. furfur* mutants defective in indole production, we generated an *Agrobacterium*-mediated random-insertional mutagenesis library and evaluated 600 individual transformants for loss of pigmentation in tryptophan-supplemented liquid mDix-mp medium. A small number of mutants with reduced pigmentation were identified (Fig. 5a). Among these, two transformants (1G1 and 2B4) could be confirmed when grown independently (Fig. 5b). While 1G1 produced red pigment after prolonged incubation, mutant 2B4 was profoundly impaired in pigment production and was therefore selected to be further investigated. Inverse PCR and amplicon sequencing confirmed a transfer DNA (T-DNA) insertion in a sulfate transporter (accession KAI3628368) (Fig. 5c, top) that is the closest orthologue of the *Saccharomyces* *cerevisiae* gene *SUL1*, which is the gene name that we use in this work. Note that this gene is mistakenly named *SUL2* in GenBank under accession KAI3628368. In the model yeast, Sul1 is a high-affinity sulfate permease responsible for sulfate uptake and regulation of endogenous activated sulfate intermediates^36^.

To confirm the phenotype of the *sul1* random insertional mutant, we generated *sul1*Δ targeted deletion mutants (Fig. 5c, bottom). The strategy of generating independent *sul1*Δ mutants was preferred over the functional complementation because random mutants can often result in an unlinked genotype and phenotype^37^, and in *Malassezia*, this cannot be determined with genetic cross and segregation analysis. Two independent *sul1*Δ targeted mutants (*sul1*Δ#1, *sul1*Δ#2) phenocopied the insertional mutant 2B4 in both in liquid and solid media when tryptophan was the sole nitrogen source (Fig. 5d–f).

**Extended Data Fig. 5:**
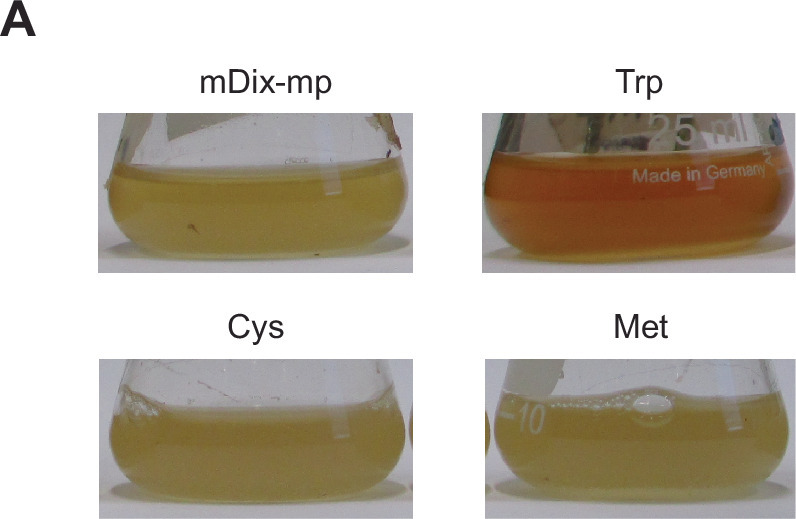
Effect of organic sulfur sources on pigmentation of *M. furfur*. **A***. M. furfur* CBS14141 grown in liquid mDix-mp supplemented with tryptophan (Trp), cystein (Cys) or methionine (Met) for 4 days.

Because Sul1 is a predicted sulfate transporter, we next tested the ability of the *sul1* mutants to use different inorganic and organic sulfur sources in the presence or absence of a nitrogen source (that is, ammonium nitrate). Compared with the parental WT, *sul1* mutants showed slightly reduced growth on MM and on MM supplemented with sulfide (Na_2_S), sulfate (NaSO_4_, MgSO_4_, KSO_4_) and the organic sulfur sources methionine (Met) and cysteine (Cys) (Fig. 5g, top), but not on potassium metabisulfite (K_2_S_2_O_5_) or sodium sulfite (NaSO_3_), indicating that *M. furfur* Sul1 is a transporter of sulfide, sulfate and the two sulfur-containing proteinogenic amino acids, but not of sulfur dioxide and sulfite. The presence of a nitrogen source did not impact the *sul1* mutant phenotypes (Fig. 5g, bottom). To correlate the observed phenotypes with the pigmentation ability of *M. furfur*, we tested the impact of the distinct sulfur sources on pigment production by growing the *M. furfur* WT in MM supplemented with tryptophan, and replacing MgSO_4_ by MgCl_2_. Sulfur sources that inhibited the growth of the *sul1* mutants (Fig. 5g) also enhanced pigment production in WT *M. furfur* (Fig. 5h,i), whereas organic sulfate sources had no impact on pigmentation (Extended Data Fig. 5a). Together, these findings link sulfur metabolism, in particular of sulfide and sulfate, to indole production.

To gain deeper insights into how Sul1 affects indole production, we compared the secretome of *sul1*Δ#1 and the parental *M. furfur* CBS14141 by untargeted metabolomics. PCA showed clustering among the different samples (Fig. 5j). Comparison of *sul1*Δ + Trp versus WT + Trp revealed 397 differential molecular features, with 142 upregulated and 255 downregulated (Supplementary Table 2), while *sul1*Δ + Trp versus *sul1*Δ without Trp resulted in 162 significant molecular features, with 79 upregulated and 83 downregulated (Supplementary Table 3). Among the detected indolic metabolites, malasseziazole C, indole-3-acetic acid, malassezin, D-indole-3-lactic acid and malasseziacitrin were found exclusively in WT + Trp, while pityriazepin/pityriacitrin and malassezindole A were detected only in the *sul1*Δ mutant (Fig. 5k). Malassezindole B, pityriazole and indole-3-carbaldehyde were present in both the *sul1*Δ mutant and WT + Trp, although malassezindole B was significantly upregulated in the mutant, indole-3-carbaldehyde was significantly downregulated and pityriazole was unchanged (Fig. 5k). No tryptophan-derived metabolites were detected in either *sul1*Δ or WT without tryptophan (Fig. 5k and Supplementary Table 4). In addition, we detected a notable accumulation of tryptophan in *sul1*Δ + Trp, whereas tryptophan levels were not detectable in WT + Trp (Fig. 5l), suggesting that Sul1 catalyses tryptophan utilization. While this does not indicate that Sul1 directly transports tryptophan, its absence may impair metabolic pathways that normally consume or process tryptophan, leading to its accumulation. In conclusion, these data indicate that most tryptophan-derived indoles in *M. furfur* depend on Sul1-mediated sulfur uptake, while the altered indole profile in the *sul1* mutant probably reflects a compensatory metabolic shift.

### Barrier integrity in inflamed skin is restored by *M. furfur*-derived indoles

**Fig. 6:**
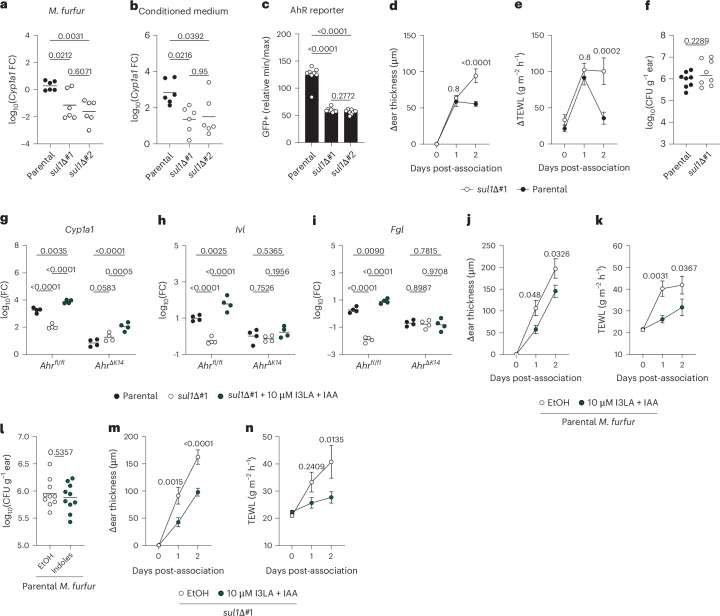
Restoration of barrier integrity in inflamed skin by *M. furfur* depends on indole production by the fungus. **a**,**b**, *CYP1A1* expression by HaCaT keratinocytes after 24 h of infection with *M. furfur sul1*Δ#1, *M. furfur sul1*Δ#2 or the WT parental strain (**a**) or exposure to conditioned medium from the same strains (**b**) grown in the presence of tryptophan. Data are plotted as the FC of +Trp relative to each strain’s −Trp baseline. *n* = 6 per group pooled from 2 independent experiments. The mean of each group is indicated. The statistical significance between mutant clones and WT was determined by one-way ANOVA. **c**, GFP expression by the reporter cell line H1G1-1C3 in response to 24 h of stimulation with conditioned medium from *M. furfur sul1*Δ#1, *M. furfur* *sul1*Δ#2 or the WT parental strain grown in the presence of tryptophan. Expression levels are shown relative to unstimulated and FICZ-stimulated cells. Each symbol represents one independently stimulated well; bars indicate the mean. *n* = 8 per group. The statistical significance of differences between mutant clones and WT was determined by one-way ANOVA. **d**–**f**, The ear skin of WT C57BL/6 mice was mildly tape stripped before epicutaneous administration of *M. furfur sul1*Δ# 1 mutant or the WT parental strain pre-grown in the presence of tryptophan. Plots show the increase in skin thickness (**d**), TEWL (**e**) and skin fungal load (**f**). *n* = 8 per group pooled from 2 independent experiments. The mean ± s.e.m. is indicated in **d** and **e**. The statistical significance between the *M. furfur* + Trp and *M. furfur* Trp groups was determined by two-way ANOVA (**d**,**e**) or unpaired two-tailed *t-*test (**f**). **g**–**i**, *Cyp1a1* (**g**), *Ivl* (**h**) and *Fgl* (**I**) expression by epidermal sheets obtained from the ear skin of *Ahr*^*fl/fl*^ or *Ahr*^Δ*K14*^ mice after 24 h of incubation in conditioned medium from *M. furfur* grown in the presence of tryptophan, either WT parental, *sul1*Δ#1 or *sul1*Δ#1 supplemented with 10 μM I3LA and IAA. Data are plotted as the FC over the mean of the respective unstimulated control. *n* = 4 per group. The mean of each group is indicated. The statistical significance of differences between groups was determined by two-way ANOVA. **j**–**l**, The ear skin of WT C57BL/6 mice was treated with I3LA and IAA in EtOH or EtOH alone for 5 consecutive days, then tape stripped until TEWL reached ~20 g m^−^^2^h^−1^ before epicutaneous administration of *M. furfur* WT parental strain pre-grown in the absence of tryptophan. **j**,**k**, Increase in skin thickness (**j**) and TEWL (**k**). **l**, Fungal load was assessed on day 2 after fungal association. *n* = 10 per group pooled from 2 independent experiments. The mean ± s.e.m. is indicated in **j** and **k**. The statistical significance of differences between the treated and EtOH groups was determined by two-way ANOVA (**j**,**k**) or unpaired two-tailed *t-*test (**l**). **m**,**n**, The ear skin of WT C57BL/6 mice was treated with 25 μl of indoles I3LA and IAA at 10 μM in EtOH or EtOH alone for 5 consecutive days, then tape stripped until TEWL reached ~20 g m^−2^ h^−1^ before epicutaneous administration of *M. furfur sul1*Δ#1 pre-grown in the presence of tryptophan. Plots show the increase in skin thickness (**m**) and TEWL (**n**). *n* = 10 per group pooled from 2 independent experiments. The mean ± s.e.m. is indicated. The statistical significance of differences between the treated and EtOH groups was determined by two-way ANOVA.

To confirm the role of indole production by *M. furfur* in the skin barrier restoration, we first assessed the consequences of Sul1 deficiency on AhR signalling. Both *sul1*Δ clones showed a strong defect in activating AhR signalling in HaCaT keratinocytes compared with WT *M. furfur* (Fig. 6a). The defect was even more pronounced when comparing the response to soluble metabolites (Fig. 6b) and confirmed using the AhR GFP reporter cell line (Fig. 6c).

We next assessed the functional consequences of impaired AhR activation by the *sul1*Δ mutant in vivo. Unlike WT *M. furfur*, *sul1*Δ grown in the presence of tryptophan failed to reduce inflammation and TEWL in tape-stripped skin (Fig. 6d,e), despite both strains colonizing the skin at comparable levels (Fig. 6f). This indicates that the phenotype was mutant intrinsic and not due to altered fungal burden.

To test whether supplementation with indoles produced by WT *M. furfur* but absent in the *sul1*Δ mutant could restore AhR-dependent barrier gene expression in the absence of a functional Sul1, epidermal sheets from *Ahr*^Δ*K14*^ and control *Ahr*^*fl/fl*^ mice were stimulated with conditioned medium from WT *M. furfur*, *sul1*Δ or *sul1*Δ supplemented with indole-3-lactic acid (I3LA) and 3-indoleacetic acid (IAA), both being commercially available. Indole supplementation restored *Cyp1a1*, *Ivl* and *Flg* expression in control but not in Ahr-deficient epidermis (Fig. 6g–i), confirming a keratinocyte-intrinsic, AhR-dependent effect. In vivo, pretreatment of skin with I3LA and IAA reduced inflammation and TEWL after colonization with WT *M. furfur* without tryptophan supplementation (Fig. 6j,k), without detectable differences in fungal colonization (Fig. 6l). Similar effects were observed with the *sul1*Δ mutant supplemented with tryptophan (Fig. 6m,n). Together, these results confirmed that indole production by *M. furfur* is essential for AhR activation in keratinocytes and for the AhR-mediated skin-protective effects of *M. furfur*.

## Discussion

As an abundant member of the skin microbiota^38^, *Malassezia* has uniquely adapted to the lipid-rich environment of the mammalian skin. Its persistence across different skin body sites suggests that it may play a role in skin physiology, yet its contributions to specific structural and immunological barrier functions have remained unexplored. In this Article, we show that *M. furfur*, via the secretion of indole metabolites, supports epidermal barrier homeostasis. Our analyses focused on *M. furfur* for mechanistic exploration of *Malassezia*-mediated AhR activation; whether other *Malassezia* species also stimulate the AhR pathway in the skin remains to be determined. The findings of our study both challenge and complement the prevailing view that *Malassezia* is an opportunistic pathogen^39^ and instead support a context-dependent role in skin homeostasis. Understanding the dual role of *Malassezia* in health and disease provides a more nuanced understanding of *Malassezia*–host interactions and emphasizes the importance of microbial-derived metabolites in maintaining skin health.

Although the detailed mechanisms of pathogenesis underlying *Malassezia*-associated skin diseases remain to be uncovered, the fungus is thought to contribute to disease progression by secreting enzymes that exacerbate barrier defects and inflammation^15,18,40–42^, as well as via allergen-driven IgE and Th2 responses in atopic dermatitis^43^. Although *M. furfur* is not the most prevalent *Malassezia* species in healthy skin, its relative abundance increases in atopic and seborrhoeic dermatitis in comparison to healthy skin^44,45^, and isolates from diseased skin have been reported to show enhanced indole production^9,10^. This led to the assumption that indole production contributes to pathogenesis^9^. Our findings, however, challenge this hypothesis and instead support a host-protective role for fungal indoles inducing AhR signalling in the host to enhance skin barrier function and dampen inflammation. Rather than driving disease, indole production may be an adaptive mechanism of the fungus that gets activated under pathological conditions to restore tissue homeostasis.

Our findings align with previous reports linking microbial and dietary indoles to barrier integrity^33,34,46^. However, in most of these studies, the microbial species responsible for indole production remained unidentified, leaving the specific microorganisms contributing to AhR activation largely speculative. By contrast, our study identifies *M. furfur* as a defined microbial source of AhR ligands in the skin. The identification of an *M. furfur* mutant showing reduced indole production, impaired restoration of barrier functions and reversion of its phenotype with I3LA and IAA confirmed that *M. furfur*-derived indoles actively contribute to AhR-mediated skin barrier homeostasis and established a direct functional link between fungal metabolism and host barrier regulation. It is also likely that the other indoles detected in *M. furfur* supernatants also contribute to AhR activation, as reported in previous studies^25,47^.

*Malassezia*’s ability to produce indoles is regulated not only by the availability of tryptophan in the stratum corneum and in sweat^19,48^, but also by that of sulfur: the phenotype of the *sul1* mutant indicates indeed that efficient indole biosynthesis depends on uptake of sulfide and/or sulfate, which probably function as cofactors of enzymes responsible for tryptophan deamination. This suggests that *Malassezia*’s metabolic output is highly adaptable and responds to specific environmental cues present in the skin. Sulfur-containing compounds are available in the skin and constantly renewed through keratin degradation, sebum components and sweat-derived thiols^49^. Similar links between sulfur metabolism and pigment production have been described in *Ustilago maydis*^50^, whereas alternative pathways exist in *Cryptococcus neoformans*, a basidiomycete related to *Malassezia* and *Ustilago*^51^. These observations suggest that the evolution of fungal tryptophan degradation pathways has been shaped by environmental factors that drive fungal adaptation. Given the abundance of sulfur-containing compounds in the skin, it is plausible that the integration of sulfur metabolism into *Malassezia*’s tryptophan metabolic pathway represents an adaptation to its host-associated lifestyle, paralleling the evolutionary adaptation of *Ustilago*, which thrives in sulfur-rich plant environments.

The anti-inflammatory consequences of AhR signalling in barrier tissues^52,53^ have led to increasing interest in AhR-targeting therapies^54^. Agents such as tapinarof^55,56^ and indirrubin^57^ corroborate the clinical relevance of AhR activation in skin inflammation^55–57^. Modulation of the *Malassezia* metabolome has been proposed as a prospective treatment strategy^58^, although the pleiotropic effects of AhR ligands require careful considerantion.

Together, our findings reveal a dynamic interplay between microbial signals and keratinocyte-specific AhR activation in shaping the skin’s barrier properties. The ability of microbial-derived metabolites, particularly fungal indoles, to modulate epidermal differentiation, immune responses and barrier restoration points at a previously underappreciated role for the skin mycobiota in maintaining homeostasis. Our mechanistic analyses focused on a single *Malassezia* species with strong AhR-activating potential and genetic accessibility, while acknowledging that other members of the genus may also contribute to cutaneous AhR signalling to varying degrees. Expanding the scope of microbiota research to include fungal-derived bioactive compounds will be critical for understanding their contributions to skin physiology and developing microbiome-targeted therapies to enhance skin health and restore barrier integrity in common inflammatory cutaneous disorders.

## Methods

### Fungal strains

All *Malassezia* strains used in this study are listed in Supplementary Table 5. *M. furfur* CBS14141 and *M. sympodialis* ATCC42132 were originally obtained from J. Heitman (Duke University Medical Center, Durham, NC). *M. pachydermatis* ATCC14522 (CBS1879) and *M. furfur* CBS1878 were purchased from ATCC. *M. restricta* CBS7877 and *M. globosa* MYA-4612 (CBS7966) were obtained from P. Bosshard (University Hospital Zurich, Switzerland). All strains were maintained at 30 °C with shaking in liquid mDixon (36 g malt extract (Sigma), 20 g desiccated Ox-bile (Sigma), 10 ml Tween-40 (Sigma), 6 g peptone (Oxoid), 2 ml glycerol (Honeywell), 2 ml oleic acid (Sigma) per 1 l) or in mDix-mp (36 g malt extract, 10 g desiccated Ox-bile, 10 ml Tween-40 and 2 ml glycerol per 1 l), before culturing in assay-specific media, as specified below and in the Results section. To quantify fungal density, cultures were washed twice and resuspended in PBS. The number of yeast cells was determined by measuring OD_600_ (1 OD_600_ = 5 × 10^6^ yeast cells per ml). For heat inactivation, fungi were incubated at 95 °C for 1 h.

### Random insertional mutagenesis of *M. furfur*

Random insertional mutants were generated by *Agrobacterium tumefaciens*-mediated transformation using the nourseothricin (*NAT*) resistance marker^24^. NAT-resistant transformants were screened for pigmentation deficiency following growth on mDixon-mp supplemented with tryptophan. Mutants showing impaired pigmentation were confirmed independently under identical growth conditions. Genomic DNA from selected strains was extracted using the CTAB method and subjected to inverse PCR to determine T-DNA insertion sites. Amplicons were Sanger sequenced and subjected to BLAST analysis against the *M. furfur* CBS14141 genome (GCA_023510035.1)^22^. The function of the mutated gene was assigned based on homology searches in GenBank and the *Saccharomyces* Genome Database.

### Targeted mutagenesis of the *M. furfur SUL1* gene

Targeted *sul1*Δ deletion mutants were generated using *A. tumefaciens*-mediated transformation^24^. The deletion construct was assembled in *S. cerevisiae* using the gap repair method, with pGI3 as backbone plasmid^59^, and with the T-DNA including a *NAT* resistance cassette flanked by 1.5-kb *SUL1* 5′ and 3′ homologous regions. The resulting plasmid was electropored into *A. tumefaciens* strain EHA105 and subsequently used to transform *M. furfur* CBS14141. NAT-resistant transformants were screened by diagnostic PCR to verify correct gene replacement. Primers used are listed in Supplementary Table 6.

Pigment production was assessed in liquid and solid mDix-mp and MM as described below. The ability to grow on different sulfur sources at 30 °C was assessed by spotting 3 µl of 1:10 dilutions of each cellular suspension on MM medium (MgSO_4_ replaced by MgCl_2_) with glucose with and without ammonium nitrate in the presence of 2 mM of these sulfur sources: Na_2_S, K_2_S2O_5_, Na_2_SO_3_, MgSO_4_ × 7 H_2_O, MgSO_4_ × 1H_2_O, Na_2_SO_4_ or KSO_4_; methionine and cysteine were used at 5 mM.

### Pigment production by *M. furfur*

To induce pigmentation and indole production, *Malassezia* strains were grown in mDix-mp, MM (15 g glucose, 1 g KH_2_PO_4_, 0.5 g KCl, 0.5 g MgSO_4_, 4 ml Tween 60, 1 ml Tween 20, 4 g Ox-bile per 1 l; MM was also tested with glucose and ammonium sulfate (3.3 g), with ammonium sulfate without glucose, and without glucose and ammonium sulfate) or Tween 80 agar (30 ml of Tween 80 for 1 l) supplemented with 5 mg ml^−1^ of L-tryptophan, if not specified otherwise. For solid media, 2% agar was added.

Subsequently, 250 µl of each cellular suspension was withdrawn daily, of which 100 µl was used to monitor growth at OD_600_ and 100 µl was centrifuged to collect cell-free supernatant for monitoring pigmentation at OD_400_; the ratio OD_400_/OD_600_ is used to express pigment production on growth.

Pigmentation of CBS14141 in the presence of different sulfur sources was carried out in MM (MgSO_4_ replaced by MgCl_2_) with and without ammonium nitrate (20 mM), and with 2 mM of the same sulfur sources used to characterize the *sul1*Δ mutant phenotypes.

### Preparation of conditioned medium

*Malassezia* strains were cultured in liquid mDixon medium for 2 days at 30 °C with shaking. A total of 2.5 × 10^7^ yeast cells were then inoculated onto Tween 80 agar plates containing or not containing 5 mg ml^−1^ L-tryptophan and incubated at 30 °C for 5 days. Fungal cells were collected using a cell scraper and resuspended in 4 ml of Ham’s F12 (Gibco) supplemented with 1% foetal calf serum and 1% penicillin/streptomycin (complete F12). For HEE experiments, yeast cells were resuspended in CnT Prime 3D Barrier medium (CELLnTEC). To remove fungal cells, the samples were passed through a 0.22-µm filter, and sterility was verified by plating on mDixon agar.

### Metabolomics

*M. furfur* CBS14141 and the *sul1*Δ#1 mutant were grown in mDix-mp with and without 0.5 mg ml^−1^ of tryptophan for 4 days at 30 °C under shaking conditions. Supernatants were filter sterilized and extracted with ethyl acetate (EtOAc, gas chromatography–mass spectrometry grade, Merck), followed by drying with anhydrous sodium sulfate (Na_2_SO_4_, Carlo Erba). The solvent was removed under vacuum at 37 °C using a rotary evaporator (RV 10, IKA-Werke). Each extract was solubilized in methanol (MeOH, LC–MS grade, Fluka) at a final concentration of 1 mg ml^−1^ and analysed by LC–MS quadrupole time of flight (Agilent Technologies). Then, 7 µl was injected into an Agilent HP 1260 Infinity Series liquid chromatograph equipped with a diode array detector and coupled to a quadrupole time-of-flight mass spectrometer. Analyses were performed according to ref. ^60^. Raw data were preprocessed using MassHunter Profinder software 8.0 (Agilent Technologies), and mass spectra were aligned and log_10_normalized for intensity before statistical analysis. Samples were categorized based on strain and/or tryptophan supplementation and subjected to PCA. Significant metabolite accumulation between groups was determined by unpaired two-tailed *t-*test with FDR correction (cut-off = 0.05) and by FC ± 2.0. Metabolite identification and annotation were performed by comparing monoisotopic data with a custom-built fungal database and previously published data^25,61–63^. Three independent cultures of each strain were prepared, and each sample was injected two times.

### Cell lines and maintenance

HaCaT cells and the AhR reporter cell line H1G1-1C3 (ref. ^27^) were maintained in DMEM high glucose supplemented with 10% foetal calf serum and 1% penicillin/streptomycin. N/TERT1 cells^64^ were cultured in Keratinocyte-Serum Free Medium (K-SFM, Invitrogen) supplemented with bovine pituitary extract (half tube supplied with K-SFM), epidermal growth factor (0.2 ng ml^−1^) and calcium chloride (0.3 mM), without antibiotics and antimycotics. All cells were incubated at 37 °C and 5% CO_2_. For routine passaging, cells were washed with PBS and, detached with 0.05% Trypsin–EDTA. Experiments were conducted with cells at passages 30–50 (HaCaT), passages 38–45 (N/TERT1) and passages 10–30 (H1G1-1C3).

### Cas9 generation of *AHR* KO N/TERT1

*AHR* knockout (KO)-N/TERT1 cells were generated according to ref. ^65^. Briefly, the 20-nucleotide gRNA targeting sequence for *AHR* was designed using the free online software ChopChop (https://chopchop.cbu.uib.no/) and Off-Spotter (https://cm.jefferson.edu/Off-Spotter/). Single-guide RNA (sgRNA) with the designed *AHR* targeting sequence and a negative control sgRNA (TrueGuide sgRNA Negative Control, non-targeting 1 (Invitrogen)) were obtained from Invitrogen and prepared according to the manufacturer’s instructions. For assembling the RNP complexes, 24 pmol sgRNA and 24 pmol TrueCut HiFi Cas9 Protein (Invitrogen) were mixed and incubated at room temperature for 20 min before adding 0.15 × 10^6^ cells in 5 μl of buffer R (10 μl total volume) and electroporated with the Neon Transfection System (Invitrogen) and the Neon Transfection System 10 μl Kit (Invitrogen), applying 1,700 V per 20-ms pulse width per pulse. The electroporated cells were seeded in a T150 flask with 30 ml complete K-SFM medium and expanded without passaging for at least a week. The medium was changed every 2–3 days. After expansion, cells were serially diluted for single-cell seeding, and individual clones were isolated.

### HEEs

N/TERT1 cells were cultured in complete K-SFM until confluency and seeded (2 × 10^5^ cells per insert) onto 0.4-µM ThinCert inserts (Greiner Bio One, 662641) in 24-well plates containing CnT Prime medium (CELLnTEC). After the cells reached confluency, the medium was replaced with 3D barrier medium, and after overnight incubation, an air–liquid interface was established by removing the medium from the insert. The medium in the lower chamber was refreshed three times per week. HEEs were used after 21 days of differentiation.

### Isolation and culture of human primary keratinocytes

Foreskin biopsies were obtained from four males undergoing routine surgical interventions (for example, surgery for phimosis) at the Doktorhaus Fällanden, Switzerland, collected with informed written consent following approval from the local ethical committee (Kantonale Ethikkommission Zürich, numbers 2015-0198 and 2024-01030) and were conducted according to the Declaration of Helsinki principles. Human primary keratinocytes (HPKs) were prepared as described^66^. Briefly, the foreskin biopsy was disinfected by a short incubation in ethanol, 5 min incubation in PBS 10% antibiotic/antimycotic (A/A, Thermo Fisher Scientific) and 5 min in PBS 1% A/A and finally washed in pure PBS. Fat was removed from the skin biopsy and the tissue was cut into small pieces. The skin pieces were then incubated for 2 h in DMEM containing 1% A/A (without FBS) and subsequently in 4 U ml^−1^ Dispase II (Roche) in PBS, overnight at 4 °C. The epidermis was separated from the dermis and incubated in 0.25% Trypsin–EDTA (Thermo Fisher Scientific) in PBS for 20 min at 37 °C. A single keratinocyte cell suspension was obtained by pipetting the epidermis up and down in DMEM containing 25% FBS and 1% A/A and passing it through a 100-μm cell strainer. Cells were centrifuged (170 × *g*, 3 min, RT) and resuspended in complete keratinocyte medium (3 parts DMEM, 1 part Ham’s F12, 10% FBS, 1% A/A, 20 μg ml^−1^ adenine (Sigma), 5 μg ml^−1^ apo-Transferrin (Sigma), 2 nM 3,3′,5-triiodo-L-thyronine (Sigma), 200 ng ml^−1^ hydrochortison (Sigma), 100 pg ml^−1^ cholera toxin (Sigma), 5 μg ml^−1^ insulin (Sigma), 10 ng ml^− 1^ EGF (Sigma)). A total of 10^6^ keratinocytes were plated on top of 2 × 10^5^ mitotically inactivated J2 feeder cells. After expansion of HPKs for two passages on feeder cells, they were cultivated without feeder cells in K-SFM medium with EGF and BPE.

### Keratinocytes and HEE infection and treatment with conditioned medium

For experiments with cell monolayers, 5 × 10^4^ cells per well were seeded in 100 µl DMEM (HaCaT, HPK, H1G1-1C3 cells) or K-SFM (N/TERT1 cells) in 96-well plates and incubated overnight at 37 °C and 5% CO_2_. The following day, the medium was refreshed, and 2 × 10^6^ yeast cells in 100 µl complete F12 medium or 100 µl of conditioned medium were added. For HEEs, 2 × 10^6^ yeast cells in 100 µl of PBS were added, and after 30 min, when the yeast cells had settled, the medium was carefully removed with a micropipette. For treating HEEs with conditioned medium, the medium in the bottom well was replaced with 1 ml conditioned medium. FICZ (1 µM) was used as a positive control for AhR activation.

### AhR reporter cell assay

After 24 h of H1G1-1C3 cell stimulation, GFP fluorescence was measured using a plate reader (Tecan) with excitation at 485 nm and emission at 515 nm. The signal from unstimulated cells was subtracted from all values, and AhR activation was expressed as a percentage relative to the FICZ-stimulated cells.

### Animals

Female WT C57BL/6j mice were purchased from Janvier Elevage and used for experiments at 6–14 weeks of age. *AhR*^−/−^ mice were generated by crossing *AhR*^*fl/fl*^ (Jax number 006203)^67^ and CMV-cre (Jax number 006054)^68^. *AhR*^*ΔK14*^ mice were generated by crossing *AhR*^*fl/fl*^ (Jax number 006203) and K14-cre^69^. Female and male (conditional) knockout mice were used at 6–14 weeks of age for experiments, and littermates were used as controls. Of note, the *AhR*^*fl/fl*^ strain was originally generated from 129-derived embryonic stem cells that carry the low-affinity *Ahr* allele (*Ahrᵈ*)^70^. Homozygous *AhR*^−/−^ mice were crossed with WT C57BL/6j, and experiments were conducted with *AhR*^−/−^ knockouts and *Ahr*^+/−^ littermates, in which the intact *Ahr* allele is derived from the C57BL/6 background bearing the high-affinity variant of the *AhR* allele (*Ahr*^*b*^).

All mice were kept at the Institute of Laboratory Animals Science, University of Zurich) in individually ventilated cages under specific pathogen-free conditions at 21–24 °C, 40–60% humidity and a standard light cycle (12 h:12 h) and were provided with unrestricted access to water and food (irradiated vitamin-fortified maintenance extrudate, Kliba Nafag number 3435). The animals were allowed to acclimatize for 1 week after arrival in the biosafety level 2 animal experimentation unit before the experiments started. Only animals in good health were included in the experiments. Experiments with WT mice used a randomized design; experiments with genetically modified mice were not done fully randomized owing to variable distribution of the different genotypes in each litter. Colonized and non-colonized animals were kept separately to avoid cross-contamination. Sample size was chosen using Fermi’s approximation and based on experience.

### Epicutaneous colonization of mouse ear skin

All mouse experiments in this study were conducted in strict accordance with the guidelines of the Swiss Animals Protection Law and were performed under the protocols approved by the Veterinary Office of the Canton Zurich, Switzerland (license number ZH142/2021 and ZH200/2024). All efforts were made to minimize suffering and ensure the highest ethical and humane standards according to the 3R principles: replace, reduce, refine^71^. Mice were anaesthetized by injection of 65 mg kg^−1^ ketamine and 13 mg kg^−1^ xylazine. The dorsal side of the ears was subjected to tape stripping using Transpore Hypoallergenic tape (3M; 5 rounds per ear) to mildly disrupt the stratum corneum. Subsequently, 10^8^ *M. furfur* suspension cells grown in mDixon and resuspended in 100 µl olive oil were applied epicutaneously to the dorsal side of each ear. Control groups received olive oil (vehicle). In some experiments, the ear skin was pretreated with 25 µl I3LA and IAA (Sigma) at 10 µM in EtOH, or pure EtOH as control, daily for 5 consecutive days^72^ before fungal colonization. Ear thickness and TEWL were measured before colonization and then daily throughout the experiment using an Oditest S0247 0–5 mm measurement device (KROEPLIN) and a Tewameter TM Nano device (Courage + Khazaka), respectively. For determining skin fungal loads, ear tissue was homogenized in water supplemented with 0.05% Nonidet P40 (AxonLab) using a TissueLyser II (Qiagen) and plated on mDixon agar for enumeration after 3–4 days of incubation at 30 °C.

### Epidermal sheet preparation and stimulation

Mouse ears were collected, and the dorsal and ventral sides were separated mechanically and enzymatically digested with 3 U ml^−1^ Dispase II (Roche) in PBS for 1 h at 37 °C before the epidermis was carefully separated from the dermis. Epidermal sheets were incubated in conditioned medium for 24 h at 37 °C and 5% CO_2_, snap-frozen in liquid nitrogen and stored at −80 °C for RNA isolation.

### CYP1A1 enzymatic activity assay (ethoxyresorufin-O-deethylase assay)

Epidermal sheets were placed in a 96-well plate containing 100 µl 2 µM 7-ethoxyresorufin (7-ERO, Sigma) in sodium phosphate buffer (50 mM, pH 8.0) per well and incubated at 37 °C for 20 min. The reaction was terminated by adding fluorescamine (150 µg ml^−1^ in acetonitrile, Sigma). Resorufin formation was quantified on a plate reader (Tecan) with 535-nm excitation and 590-nm emission wavelengths using a resorufin (Sigma) standard curve, as described^73^. After termination of the assay, epidermal sheets were snap-frozen in liquid nitrogen and stored at −80 °C for RNA isolation.

### Neutrophil quantification

Ears were collected from colonized mice, cut into small pieces and enzymatically digested in Ca^2+^- and Mg^2+^-free Hank’s medium (Life Technologies) supplemented with Liberase TM (0.15 mg ml^−1^, Roche) and DNase I (0.12 mg ml^−1^, Sigma) for 1 h at 37 °C. Single-cell suspensions were obtained by mechanical dissociation using a 70-µm cell strainer. Cells were stained with AF700 anti-CD45.2 (clone 104, Biolegend), Pacific Blue anti-Ly6G (clone 1A8, Biolegend) and PE-Cy5 anti-CD3ε (clone 145-2C11, Biolegend) at 4 °C for 30 min in the dark. LIVE/DEAD Fixable Near-IR stain (Life Technologies) was used to exclude dead cells. Flow cytometry was performed using a CytoFLEX S (Beckman). Data were analysed with FlowJo software. Gating strategies followed the guidelines for the use of flow cytometry and cell sorting in immunological studies^74^, including pre-gating on viable and single cells (Extended Data Fig. 3e).

### Histology

Mouse tissues were fixed in 4% PBS-buffered paraformaldehyde overnight and embedded in paraffin. Sagittal sections (9 µm) were stained with periodic acid–Schiff reagent, followed by counterstaining with haematoxylin. Slides were mounted using Pertex mounting medium (Biosystem) according to standard protocols. Histological images were acquired using a digital slide scanner (NanoZoomer 2.0-HT, Hamamatsu) and analysed with NDP.view2 software. Epidermal thickness was measured at five randomly selected areas per image, each separated by 1 µm along the epithelium using the ruler tool of the NDP.view2 software.

### RNA isolation and RT-qPCR

Isolation of total RNA was done using TRI-Reagent (Sigma) according to the manufacturer’s instructions. For adherent cells, cells from 4 wells of a 96-well plate were pooled in 500 µl TRI-Reagent. Mouse epidermal sheets and HEE samples were collected in 500 µl TRI-Reagent and homogenized using TissueLyzer II for 6 min at 25 Hz. RNA was isolated by phase separation with chloroform, precipitated with isopropanol, washed in 75% EtOH, air-dried and resuspended in ultra-pure H_2_O. cDNA was generated by RevertAid reverse transcriptase (Thermo Fisher) and random nonamers. qPCR was performed using SYBR green (Roche) and a 7500 Real-Time PCR System (Applied Biosystems) instrument. The primers are listed in Supplementary Table 7. All RT-qPCR assays were performed in duplicates, and the relative expression of each gene was determined after normalization to mouse *Actb* or human *G6CPD* housekeeping genes, respectively. Data are shown as fold change, which was calculated by dividing the relative expression of each sample in the treated group by the average of the control group, and then log transformed.

### Conventional PCR

The excision of *Ahr*^fl/fl^ was assessed by PCR using the forward primers OL4062 and OL4064, along with the reverse primer OL4088 (Supplementary Table 8). The PCR products allowed differentiating between the excised allele (OL4062/OL4088, 180 bp), the unexcised allele (OL4064/OL4088, 140 bp) and the WT allele (OL4064/OL4088, 106 bp).

### Statistical analysis

All statistical analyses were performed using GraphPad Prism version 10.4.1 for Windows (GraphPad Software, www.graphpad.com): unpaired two-tailed *t*-test was used when comparing two groups; one-way ANOVA followed by Tukey’s multiple-comparison test was used for experiments involving more than two groups and a single independent variable, and two-way ANOVA followed by Sidak’s multiple-comparison test was applied when analysing the effect of two independent variables. For metabolomics data, statistical analyses were done using Mass Profiler Professional (version 13.1.1, Agilent Technologies) and MetaboAnalyst version 6.0 (https://www.metaboanalyst.ca/). Data visualization was conducted using GraphPad Prism and ggplot2 within the R statistical environment (Rstudio version 2024.12.0). Details of each analysis, including specific statistical analyses and *P* values, are provided in the figure legends.

### Reporting summary

Further information on research design is available in the Nature Portfolio Reporting Summary linked to this article.

## Supplementary information

Reporting Summary

Peer Review File

Supplementary Tables Supplementary Tables 1–8.

## Data Availability

Data supporting the findings of this study are available via Zenodo at 10.5281/zenodo.19630847 (ref. ^75^).
