# Supplementary material for: Commensal yeast Malassezia produces tryptophan metabolites to promote tissue homeostasis via the aryl hydrocarbon receptor in mice
Source: Nat Microbiol. 2026 Jun 19;11(7):1934–48. doi: 10.1038/s41564-026-02382-8 (PMC13323057; doi:10.1038/s41564-026-02382-8)
Supplement: Supplementary file 1 — Reporting Summary [file 41564_2026_2382_MOESM1_ESM.pdf]

nature portfolio

Corresponding author(s): Salomé LeibundGut-Landmann

Last updated by author(s): Apr 14, 2025

Reporting Summary

Nature Portfolio wishes to improve the reproducibility of the work that we publish. This form provides structure for consistency and transparency in reporting. For further information on Nature Portfolio policies, see our Editorial Policies and the Editorial Policy Checklist.

Statistics

For all statistical analyses, confirm that the following items are present in the figure legend, table legend, main text, or Methods section.

n/a	Confirmed	
☐	☒	The exact sample size (*n*) for each experimental group/condition, given as a discrete number and unit of measurement
☐	☒	A statement on whether measurements were taken from distinct samples or whether the same sample was measured repeatedly
☐	☒	The statistical test(s) used AND whether they are one- or two-sided<br>*Only common tests should be described solely by name; describe more complex techniques in the Methods section.*
☒	☐	A description of all covariates tested
☒	☐	A description of any assumptions or corrections, such as tests of normality and adjustment for multiple comparisons
☐	☒	A full description of the statistical parameters including central tendency (e.g. means) or other basic estimates (e.g. regression coefficient) AND variation (e.g. standard deviation) or associated estimates of uncertainty (e.g. confidence intervals)
☐	☒	For null hypothesis testing, the test statistic (e.g. *F*, *t*, *r*) with confidence intervals, effect sizes, degrees of freedom and *P* value noted<br>*Give P values as exact values whenever suitable.*
☒	☐	For Bayesian analysis, information on the choice of priors and Markov chain Monte Carlo settings
☒	☐	For hierarchical and complex designs, identification of the appropriate level for tests and full reporting of outcomes
☒	☐	Estimates of effect sizes (e.g. Cohen's *d*, Pearson's *r*), indicating how they were calculated

Our web collection on statistics for biologists contains articles on many of the points above.

Software and code

Policy information about availability of computer code

Data collection	RTqPCR: ABI7500Fast; scanning of histology slides: NanoZoomer 2.0-HTdigital scanner; flow cytometry: CytoFlex S; metabolomics: LC–MS qTOF
Data analysis	GraphPad Prism v10 was used to create graphs and performed statistical analyses; FlowJo v10 was used to analyse flow cytometry data; ABI7500 software v2.3 was used to analyse gene expression data; NDP. view version 2 was used to analyse histology images; MassHunter Profinder software version 8 were used to analyse metabolomic data.

For manuscripts utilizing custom algorithms or software that are central to the research but not yet described in published literature, software must be made available to editors and reviewers. We strongly encourage code deposition in a community repository (e.g. GitHub). See the Nature Portfolio guidelines for submitting code & software for further information.

Data

Policy information about availability of data

All manuscripts must include a data availability statement. This statement should provide the following information, where applicable:

- Accession codes, unique identifiers, or web links for publicly available datasets
- A description of any restrictions on data availability
- For clinical datasets or third party data, please ensure that the statement adheres to our policy

All raw data linked to this study are publically available on Zenodo (doi: 10.5281/zenodo.19630847)

Research involving human participants, their data, or biological material

Policy information about studies with human participants or human data. See also policy information about sex, gender (identity/presentation), and sexual orientation and race, ethnicity and racism.

Reporting on sex and gender	Human cells were isolated from foreskin, i.e. male.
Reporting on race, ethnicity, or other socially relevant groupings	unknown
Population characteristics	n/a
Recruitment	n/a
Ethics oversight	n/a

Note that full information on the approval of the study protocol must also be provided in the manuscript.

Field-specific reporting

Please select the one below that is the best fit for your research. If you are not sure, read the appropriate sections before making your selection.

☒ Life sciences ☐ Behavioural & social sciences ☐ Ecological, evolutionary & environmental sciences

For a reference copy of the document with all sections, see nature.com/documents/nr-reporting-summary-flat.pdf

Life sciences study design

All studies must disclose on these points even when the disclosure is negative.

Sample size	No statistical methods were used to pre-determine sample sizes but our sample sizes are similar to those reported in previous publications (e.g., Sparber et al. 2019, PMID: 30870621).<br>Sample sizes were chosen by the Fermi's approximation and based on experience. We generally used 3-5 mice per group and experiment. Experiments were generally repeated at least 2 times and data were pooled.
Data exclusions	Datapoints determined as outliers based on an outlier analysis performed with GraphPad Prism were excluded.
Replication	Experiments were generally repeated at least 2 times. All replications were successful.
Randomization	Experiments with C57BL/6JRj WT mice used a randomized design. Experiments with genetically modified animals were not done fully randomized due to variable distribution of the different genotypes in each litter. Upon weaning, mice were caged by sex, but not genotype. Animals were allocated to experimental groups based on genotype while accounting for sex, age, and litter where possible. Experimental groups were kept separate during the experiment to prevent fungal transmission between groups.
Blinding	Mouse infections and treatments could not be carried out fully blinded. Turbidity of the fungal inoculum inadvertently informed the experimenter about the identity of the infection vs. the control inoculum. TEWL and ear thickness measurements were coded to blind the experimenter to genotype. Sample collection during the experiment and ex vivo analyses such as counting of fungal colonies, qPCR and analysis of histology sections were executed in a blinded way wherever possible by using coded numeric identifiers for samples throughout processing and data acquisition. Ex vivo flow cytometry analyses were done in an unbiased way through applying a fixed gating strategy to all samples of the experiment.

Reporting for specific materials, systems and methods

We require information from authors about some types of materials, experimental systems and methods used in many studies. Here, indicate whether each material, system or method listed is relevant to your study. If you are not sure if a list item applies to your research, read the appropriate section before selecting a response.

Materials & experimental systems

n/a	Involved in the study
☐	☒ Antibodies
☐	☒ Eukaryotic cell lines
☒	☐ Palaeontology and archaeology
☐	☒ Animals and other organisms
☒	☐ Clinical data
☒	☐ Dual use research of concern
☒	☐ Plants

Methods

n/a	Involved in the study
☒	☐ ChIP-seq
☐	☒ Flow cytometry
☒	☐ MRI-based neuroimaging

Antibodies

Antibodies used	All antibodies were purchased from BioLegend: CD45.2-AlexaFluor700 (clone 104, Biolegend #109822, dilution 1:700); CD3-PE-Cy5 (clone145-2C11, Biolegend #100310, dilution 1:700); Ly6G-Pacific Blue (clone 1A8, Biolegend #127612, dilution 1:300)
Validation	All the antibodies were validated by the manufacturer.<br>Ly6G: https://www.biolegend.com/fr-ch/products/pacific-blue-anti-mouse-ly-6g-antibody-6082<br>CD45.2: https://www.biolegend.com/fr-ch/products/alexa-fluor-700-anti-mouse-cd45-2-antibody-3393<br>CD3e:https://www.biolegend.com/fr-ch/products/pe-cyanine5-anti-mouse-cd3epsilon-antibody-26

Eukaryotic cell lines

Policy information about cell lines and Sex and Gender in Research

Cell line source(s)	HaCaT cells were provided by Sabine Werner, N/TERT1 were available in H.D. Beer's lab (originally provided by James G. Rheinwald); H1G1-1C3 were provided by Michael S. Deninson.
Authentication	HaCaT: RRID:CVCL_0038; N/TERT: RRID:CVCL_CW92; H1G1-1C3: RRID:CVCL_DC19. Cell lines were not authenticated.
Mycoplasma contamination	The cell lines were not tested for mycoplasma contamination.
Commonly misidentified lines<br>(See ICLAC register)	n/a

Animals and other research organisms

Policy information about studies involving animals; ARRIVE guidelines recommended for reporting animal research, and Sex and Gender in Research

Laboratory animals	WT C57BL/6Jj mice were purchased from Janvier Elevage and used at 6-14 weeks of age.<br>AhR−/− mice were generated by crossing AhRfl/fl (Jax #006203) and CMV-cre (Jax #006054) and then backcrossed to C57Bl/6j. Experiments were conducted with AhR-/- vs. heterozygous littermate controls at 6-14 weeks of age.<br>AhRΔK14 mice were generated by crossing AhRfl/fl (Jax #006203) and K14-cre. Experiments were conducted with K14-cre+ vs. K14-cre- animals, all of which carried two mutant AhR alleles, at 6-14 weeks of age.<br>All mice were kept in individually ventilated cages under specific pathogen-free conditions at 21 - 24°C, 40-60% humidity, and a standard light cycle (12h:12h) and were provided with unrestricted access to water and food (irradiated vitamin-fortified maintenance extrudate, Kliba Nafag #3435). Animals were allowed to acclimatize for 1 week after arrival in the BSL2 animal experimentation unit before starting experiments. Only animals in good health were included in experiments.
Wild animals	n/a
Reporting on sex	Experiments with WT C57BL/6Jj mice were conducted with females. Experiments with all transgenic mice and respective heterozygous littermate control mice were performed with female and male mice.
Field-collected samples	The study didn't involve field-collected samples
Ethics oversight	All mouse experiments in this study were conducted in strict accordance with the guidelines of the Swiss Animals Protection Law and were performed under the protocols approved by the Veterinary office of the Canton Zurich, Switzerland (license number ZH142/2021). All efforts were made to minimize suffering and ensure the highest ethical and humane standards according to the 3R principles

Note that full information on the approval of the study protocol must also be provided in the manuscript.

Plants

Seed stocks	n/a
Novel plant genotypes	n/a
Authentication	n/a

Flow Cytometry

Plots

Confirm that:

☒ The axis labels state the marker and fluorochrome used (e.g. CD4-FITC).

☒ The axis scales are clearly visible. Include numbers along axes only for bottom left plot of group (a 'group' is an analysis of identical markers).

☒ All plots are contour plots with outliers or pseudocolor plots.

☒ A numerical value for number of cells or percentage (with statistics) is provided.

Methodology

Sample preparation	Ears were collected from colonized mice three days post fungal association, cut into small pieces, and enzymatically digested in Ca^{2+}- and Mg^{2+}-free Hank's medium (Life Technologies) supplemented with Liberase TM (0.15 mg/mL, Roche) and DNase I (0.12 mg/mL, Sigma-Aldrich) for 1 hour at 37°C. Single-cell suspensions were obtained by mechanical dissociation using a 70 µm cell strainer (Falcon). For neutrophil identification, cells were stained with AF700 Anti-CD45.2 (clone 104), Pacific Blue anti-Ly6G (clone 1A8), and PE-Cy5 anti-CD3ε (clone 145-2C11) at 4°C for 30 minutes in the dark. LIVE/DEAD Fixable Near-IR stain (Life Technologies) was used to exclude dead cells.
Instrument	CytoFLEX (Beckman-Coulter)
Software	FlowJo v10 software (FlowJo LLC)
Cell population abundance	The abundance of the cell populations of interest is provided in Figure 3D Absolute cell numbers are provided in Figure 3E.
Gating strategy	Quantification of neutrophiles in ear skin tissue<br>SSC-Area/FSC-Area to select for all tissue cells, FSC-Heigt/FSC-Area to select single cells, CD45.2-alexa Fluor 700-Area/ live dead APC-cy7-Area to selected live immune cells, Ly6G-Pacific Blue-Area to select neutrophils.

☒ Tick this box to confirm that a figure exemplifying the gating strategy is provided in the Supplementary Information.
