# Supplementary material for: Commensal yeast Malassezia produces tryptophan metabolites to promote tissue homeostasis via the aryl hydrocarbon receptor in mice
Source: Nat Microbiol. 2026 Jun 19;11(7):1934–48. doi: 10.1038/s41564-026-02382-8 (PMC13323057; doi:10.1038/s41564-026-02382-8)
Supplement: Supplementary file 2 — Peer Review File [file 41564_2026_2382_MOESM2_ESM.pdf]

nature portfolio

Peer Review File

Commensal yeast Malassezia produces tryptophane metabolites to promote tissue homeostasis via the aryl hydrocarbon receptor in mice

Corresponding Author: Professor Salomé LeibundGut-Landmann

Version 0:

Reviewer comments:

Reviewer #1

(Remarks to the Author)
The manuscript by Gushiken-Ibañez investigates the role of Malassezia furfur in maintaining epidermal integrity. Using a fungal mutant which is not able to produce tryptophan-derived indole metabolites that activate the aryl hydrocarbon receptor (AHR) they show that M. furfur-derived AHR activation restores barrier function and controls inflammation in a keratinocyte intrinsic manner. AHR-deficient mice do not benefit from M. furfur-mediated barrier protection, highlighting the importance of microbial derived AHT activation in skin homeostasis. The novel an important finding presented here build on a growing body of evidence affirming a central role for AHR signalling in skin and provide a new perspective on the functional role of M. furfur, proposing that its metabolic activity is a compensatory response to environmental or host-derived stressors rather than a pathogenic agent in inflammatory disease as atopic dermatitis.
The manuscript is very well written, the experiments are well executed with appropriate controls used throughout, and validation experiments performed where necessary.
Nonetheless, a couple of points deserve the authors' attention:
1) AHR activation is only measured at 1 time point (24h); how is CYP1A1 expression at earlier time points? This assessment is of importance also given the next point.
2) The observation that M. furfur alone as well as the other strains i elicited a basal AHR activation independently of tryptophan (Fig. 1) is likely due to the presence of Trp in the culture media. The authors should please address /acknowledge this point.

Reviewer #2

(Remarks to the Author)
I have read with extreme interest the manuscript entitled 'The skin commensal yeast Malassezia promotes tissue homeostasis via the aryl hydrocarbon receptor'. The Authors have performed an exhaustive investigation on the interaction of 2 Malassezia furfur species with a variety of skin equivelants (cell cultures, skin equivelants, experimental animals). Their findings significanlty impact our future approach on the interaction of this ubiquitous yeast with the skin and associated skin diseases. Some points that need to be addressed are:
Major points:
There appears to be a selective presentation of statistical analysis in graphs of all figures. There are instances where specific comparisons and their statistical testing is completely ignored.
Also SEM or SD is missing from most figures. Statistical test used should be included in the figure legend, as it is key information, for each subpanel of the figure so the reader doesn't have to go back and forth in the manuscript to find this info.

All figures:

1. Figure 1:

1C. The OD value of the -Trp pigmentation should be added to the plot.
1E label the top most up or down regulated metabolites. Indicate the metabolites of interest on the volcano plot.
Number of samples should be included in Figure legend.
1G-1J: SEM is missing.
All plots should be rederived and show fold change over the control not relative expression as this is difficult to assess and the way it is calculated is not presented in the methods.
Statistics should be repeated based on fold change. Statistical test used should be clearly shown for each subpanel in the corresponding figure legend.
Figure legend for 1G-J is using the wrong letters it is referring to A-D.

2. Figure 2:

2A and 2B change to fold change
Add SEM
Indicate statistical test

2B. AHR DK14 relative expression for Ovol1 and Ivl appears statistically significant based on the data presented. Is it significantly different? if so is this reflecting a baseline effect of the Ahr KD14?

3. Figure 3

3B.Would like to see ear thickness of AHR+/- +M furfur +/- Trp on the same figure panel.

3C. SEM missing. Also include test used.

3C and 3A ear thickness measurements don't agree.
3D % of gated populations should be shown. Otherwise no conclusion can be derived from the shown representative plots.

3F. Stats missing for Ahr -/- day 1. Why does day 1 for Ahr-/- differs from Ahr+/- day 1? Surprisingly the vehicle control has a remarkably similar pattern although the actual measurements appear different.

4. Figure 4

4B. Ahrfl/fl and Ahrk14 should also be included in the same Figure panel.

4C. SEM and test used.

4D day 1 M Furfur +Trp is statistically different ?

5. Figure 5

5A. Change to fold change.
n-=2 for some groups. How were stats performed?

In Results words such as robust, marked etc should be accompanied by the corresponding p values.

Minor concerns:

A small number of typographical errors such as line 194, "possesses a enhanced "

Lines 114-115: 'This effect was mediated....indoles'. The sentence should be rephrased for clarity
Line 127: I am not certain if these two references are appropriate for describing aneuploidy of the strain. Maybe they should be moved after word 'tryptophan'
Line 145: I could not locate the heat inactivations protocol for Malassezia
Line 212-213: Reference 47 might be wrong. Please check.
Line 436-444: The findings of the Authors could be corroborated by the therapeutic use of indirubin in psoriasis (Lin et al Br J Dermatol 2018 Jan;178(1):124-131. doi: 10.1111/bjd.15894. Epub 2017 Dec 14) and the suggestion of the role of Malassezia metabolome in this disease (Gaitanis et al. Br J Dermatol
. 2018 Sep;179(3):800. doi: 10.1111/bjd.16807. Epub 2018 Jul 4.)

Reviewer #3

(Remarks to the Author)
Comments to Authors:
“The skin commensal yeast Malassezia promotes tissue homeostasis via the aryl hydrocarbon receptor.". Gushiken-Ibañez et al Malassezia Indoles Nat 32951

A. What are the noteworthy results?
The authors present a pivotal, novel, and well executed exploration of a mutualistic, host targeted Malassezia/Host interaction. Recent advances in technology have driven a renewed interest in Malassezia and Malassezia/Host interaction, particularly driven by their ubiquitous presence on mammalian skin. This manuscript presents a significant contribution to this literature and the basic biology of human skin.
The authors show that specific Malassezia species produce a wide range of tryptophan dependent metabolites and pigments, the impact of these metabolites on human skin homeostasis and health, and define the specificity by disruption thereof to abrogate the impact. They also demonstrate that Malassezia regulate us, their human host, through specific secretory molecules that drive host gene expression and physiology. This represents a breakthrough in human skin microbiome research with broad implications for human biology and disease therapy.

B. Will the work be of significance to the field and related fields? How does it compare to the established literature? If the work is not original, please provide relevant references.
The work is highly significant in skin microbiome as it is one of the first to show that skin microbes directly regulate human physiology, and in a mutualistic way. The newly discovered potential for Malassezia to play a role in multiple, non-skin diseases, such as pancreatic cancer and Crohn's Disease, expand the implications. While unmentioned by the authors (and which should be), Malassezia also contributes significant morbidity through septic infection, specifically and often M furfur.

C. Does the work support the conclusions and claims, or is additional evidence needed?
The authors present a detailed and well controlled series of experiments which clearly show that secreted Malassezia metabolites play a key role in maintenance of skin homeostasis. However, the specific molecules are not clearly defined and could be of other classes than discussed. It is abundantly clear that the tryptophan-dependent metabolites are actively produced, secreted, and dependent on tryptophan, sulfur, and sul1. However, I did not see where any of the specific molecules proposed were tested to see if they are indeed the actual molecule. This would be useful information, and if it is not one of the listed molecules it in no way detracts from the work to discuss what the specific material may be and next steps to define it. Also, the authors should clarify this throughout the manuscript.
I do not fully appreciate the inclusion of the experiments regarding metabolite production on different carbon and nitrogen sources. This confirms prior work (Mayser et al), and seems a bit out of place. This section could be significantly abbreviated and replaced with important and insufficient discussion of the skin microbial community and the choice of M furfur as a model organism.

D. Are there any flaws in the analysis, interpretation, and conclusions? Do these prohibit publication or require revision?
M furfur is now considered to be rare on human skin. Do any of the more skin relevant species (restricta, arunalokei, globosa, sympodialis) produce indoles? Please describe why M furfur is useful as a model organism.
CBS 1878 is isolated from human skin (seborrheic dermatitis), but 14141 is a septic isolate. Please discuss the implications.
How much tryptophan would be expected on human skin? How does this compare to the amount used in the culture medium? Same for sulfur.
The indole secretion and AHR activation seems limited to M furfur. The authors should be clear throughout that this may be limited to M furfur in vivo. Please add commentary.
What is the relevance of the different nitrogen/carbon source experiments? Which is most relevant to skin?
Were any of the detected metabolites, or a mixture thereof, able to induce AHR signaling? If so, which ones? If not any of those cited, what could be the activator?
It will be important to update the number, apparent biomass, and species of Malassezia found on skin. This will only reenforce the importance.
This is not the first description of a potentially mutualistic effect of Malassezia. This has been proposed in https://doi.org/10.1038/nmicrobiol.2016.106 and several including https://doi.org/10.1016/j.jid.2017.11.034 and https://doi.org/10.1073/pnas.2212533119. This must be corrected, and in general it is not appropriate to claim work to be "the first ever". Remove all instances of this claim.

E. Details:
• Typo in the abstract line 40, should be "remains"
• Overall, the manuscript could use a detailed editorial review to correct the English usage. This would substantially shorten the manuscript and improve the readability.
• Line 88: I do not believe that M furfur is among the most abundant Malassezia on human skin. The authors should consider a more recent skin microbiome review. Note that now M arunalokei is considered among the most abundant.
• Line 93: Substantial evidence implicates Malassezia as causative in seborrheic dermatitis and antifungal therapy is an accepted and effective treatment. Correct and appropriately reference this paragraph through line 101.
• Line 315: correct typo in "transformatns"
• There are numerous typos throughout, such as line 344
• You did not mention the source of all of the fungal strains.
• Define the abbreviation HPK on first usage.
• The methods section on mutant generation could be abbreviated by referencing prior work.

Reviewer #4

(Remarks to the Author)
In this manuscript by Gushiken-Ibañez et al., the investigators have explored the role of the aryl hydrocarbon receptor (AHR) in sensing Malassezia furfur in human (in vitro) and mouse (in vitro and in vivo) settings. The investigators find that M. furfur metabolizes tryptophan into a variety of metabolites that stimulate keratinocytes via the AHR and that this helps promote epidermal health and healing. These findings are curious in that they contrast with the general idea that M. furfur can be pathogenic by observing that, under the right circumstances, the organism might be supportive. Previous studies have noted indole-containing tryptophan derived production by Malassezia and activation of AHR in vitro. This study is novel in the context of looking specifically at keratinocytes, employing an in vivo skin inflammation model using AHR knockout animals, and employing a genetic screen to identify at least one M. furfur gene that is important for optimal tryptophan metabolite production. The effect of the AHR knockout in the mouse skin inflammation model is especially compelling. Overall, it is an exciting study. There are, however, a handful of shortcomings as presented.

Main points:

Figure 2 relies on using an air-liquid interface culture of the N/TERT1cell line to establish that the M. furfur tryptophan metabolites upregulate a collection of genes associated with skin barrier function. Were these genes also regulated in the Figure 1 culture of the same cell line (albeit in liquid culture) where CYP1A1 only was assessed? Are both culture approaches necessary or informative? Or is this duplicative? Also, the investigators are stimulating here with crude concoctions. The "soluble tryptophan metabolites" the authors claim to be using (for example line 204) is just the culture supernatant, which certainly contains tryptophan metabolites but also contains much more. The conclusion is likely correct, but it would be enhanced by showing that pure forms of the tryptophan metabolite are sufficient to the effects.

In a related observation, is the "organotypic human epidermal equivalents (HEEs)" really the correct language to present to readers? Even if the "HEE" bit is published language? Isn't this just air liquid interface culture of the N/TERT1cell line in transwells? This is the same cell line used in Figure 1. The language seems to suggest some kind of primary culture or "organoid" like culture, which this is not.

The conclusion that M. furfur "uniquely" induces AHR is incompletely supported by figure 1 and its supplemental data. Culture conditions were screened and optimized for pigment production by M. furfur, and then the resulting conditions were tested with single strains of other Malassezia spp. Would we get the same outcome, for example, if we had optimized the culture conditions for pigment production by an assortment of M. sympodialis strains? To strongly make this statement, further data are required. More culture conditions? More isolates? A genetic explanation of the difference? Otherwise, the conclusion needs to be couched more carefully as a preliminary indication.

Commonly in microbial knockout experiments, the control is to use of a complemented strain. Is there a technical reason that this is not realistic in the Sul1 mutant? If so, this should be explained to readers, and the alternative approach used (two independent knockouts) should be justified.

Figure 6A&B are the "-Trp" samples different from each other? Does the n need to be larger to even these out? How does the interpretation change if the Sul1 knockout produces a different level of AHR induction even in the absence of Trp? In a related observation, statistics need to be consistently described throughout. Is the cutoff designated as "significant" the same in all figures? Is the lack of a comparison shown always meant to be interpreted as "not significant"? This is especially sticky in figures like 6A where the -Trp samples look to be quite different from each other.

Figure 6D-E is incomplete. The investigator need to provide evaluation of colonization efficiency and comparison of the -/+ Trp cultures as evaluated in Figure 3. What would it mean if the ability of Malassezia to proliferate on skin is elevated in the Sul1 knockout? What would it mean if the effects of Trp are still present in the knockout?

Minor points:
The abstract describes the observation as
Figure 1G-J is reported as measuring AHR activation, but this is really measuring CYP1A induction in a couple of cell lines. This is likely to be a measure of AHR activation, but we can't rule out that something else is doing this. Receptors like LXRα and PPARα also regulate CYP1A. In the next figure the authors will get to testing this specificity in one of the cell lines with a knockout and in cells from a mouse knockout, so the conclusions here should avoid definitive language and be described as "suggestive of" or "consistent with" the idea of AHR activation until the knockouts are employed.

Growth media should be added to the resource table.

Labeling of 1D would be clearer if the right image labeled "Trp" is labeled "(-)" or such. Trp is in all the cultures, as indicated by the top label.

Line 234 – at the first mention of Ahr/K14 mice, a reference should be provided. The data would further benefit from some sort of internal control for the presence of similar numbers of keratinocytes in the compared samples – perhaps mRNA of a keratinocyte marker?

Is there a reason that fig S3A does not include the Ahr knockout? This would have confirmed that the observed Cyp1a regulation indeed is Ahr dependent.

The discussion states "we uncover a new biochemical pathway in Malassezia that controls the production of indole metabolites." Does the study really do this? There is an observation that a gene called Sul1 is important and that this suggests a role for a sulfide/sulfate source in permitting tryptophan metabolite production. But this not yet a "new pathway". What is the pathway by which a sulfur source is relevant?

In figure 2B, why is the comparison between WT and KO not shown? Is there a technical reason why this is not realistic given that the question being asked is "does the induction of Cyp1a1 by +tryp conditioned media depend on AHR?"

Lines 459-464: "Interestingly, while tryptophan metabolism is essential for indole biosynthesis across multiple fungal species, key enzymes previously implicated in indole production in other fungi, such as the aminotransferase TAM1/ARO8/AroH/ mediating the first step of tryptophan degradation in U. maydis, C. glabrata and Aspergillus fumigatus, respectively, [72-74], appear to be dispensable or redundant with other aminotransferases in M. furfur, despite a biochemical confirmation on M. furfur CBS 1878 [75]." This is a bit garbled. And to my reading, reference 75 does not suggest that MfTam1is dispensable in M. furfur.

Decision Letter:

2nd June 2025

*Please ensure you delete the link to your author homepage in this e-mail if you wish to forward it to your co-authors.

Dear Salomé,

Thank you for your patience while your manuscript "The skin commensal yeast Malassezia promotes tissue homeostasis via the aryl hydrocarbon receptor." was under peer-review at Nature Microbiology. It has now been seen by 4 referees, whose expertise and comments you will find at the end of this email. Although they find your work of potential interest, they have raised a few concerns that will need to be addressed before we can consider publication of the work in Nature Microbiology.

In particular, they have concerns with the statistical methods used, exaggeration of certain claims, and a lot of claims/methods/assertions that require further clarifications. Therefore, we'd suggest reassessing the data using appropriate statistical methods to ensure there is on bias. We highly recommend that you determine which Tryptophan metabolites are involved in inducing AHR signalling and repeat some work with purified metabolites as suggested by R3 and R4. In addition to that, we'd also suggest explaining why M. furfur, a rather rare species, was chosen and recommend strengthening your conclusion on 'uniqueness' of M. furfur to activate this signalling cascade by using other species of Malassezia and making genomic comparisons. Apart from this, in the revised version please provide the additional data as requested by R4. However, for us to consider a revised version, all the concerns, not just the ones highlighted, should be addressed in the revised version.

We are committed to providing a fair and constructive peer-review process. Please do not hesitate to contact us if there are specific requests from the reviewers that you believe are technically impossible or unlikely to yield a meaningful outcome.

We strongly support public availability of data. Please place the data used in your paper into a public data repository, if one exists, or alternatively, present the data as Source Data or Supplementary Information. If data can only be shared on request, please explain why in your Data Availability Statement, and also in the correspondence with your editor. For some data types, deposition in a public repository is mandatory - more information on our data deposition policies and available repositories can be found at https://www.nature.com/nature-research/editorial-policies/reporting-standards#availability-of-data.

Please include a data availability statement as a separate section after Methods but before references, under the heading "Data Availability". This section should inform readers about the availability of the data used to support the conclusions of your study. This information includes accession codes to public repositories (data banks for protein, DNA or RNA sequences, microarray, proteomics data etc…), references to source data published alongside the paper, unique identifiers such as URLs to data repository entries, or data set DOIs, and any other statement about data availability. At a minimum, you should include the following statement: "The data that support the findings of this study are available from the corresponding author upon request", mentioning any restrictions on availability. If DOIs are provided, we also strongly encourage including these in the Reference list (authors, title, publisher (repository name), identifier, year). For more guidance on how to write this section please see: http://www.nature.com/authors/policies/data/data-availability-statements-data-citations.pdf

If revising your manuscript:

* Include a "Response to referees" document detailing, point-by-point, how you addressed each referee comment. If no action was taken to address a point, you must provide a compelling argument. This response will be sent back to the referees along with the revised manuscript.

* If you have not done so already we suggest that you begin to revise your manuscript so that it conforms to our Article format instructions at http://www.nature.com/nmicrobiol/info/final-submission. Refer also to any guidelines provided in this letter.

* Include a revised version of any required reporting checklist. It will be available to referees (and, potentially, statisticians) to aid in their evaluation if the manuscript goes back for peer review. A revised checklist is essential for re-review of the paper.

When submitting the revised version of your manuscript, please pay close attention to our href="https://www.nature.com/nature-portfolio/editorial-policies/image-integrity">Digital Image Integrity Guidelines. and to the following points below:

-- that unprocessed scans are clearly labelled and match the gels and western blots presented in figures.
-- that control panels for gels and western blots are appropriately described as loading on sample processing controls
-- all images in the paper are checked for duplication of panels and for splicing of gel lanes.

EXTENDED DATA FIGURES

When re-submitting your manuscript, please ensure that any supplementary figures and tables that are crucial to the manuscript's conclusions are converted into Extended Data figures and tables to increase visibility of these data. Extended Data figures and tables are online-only (present in the online PDF and full-text HTML versions of the paper), peer-reviewed display items that provide essential background to the article but are not included in the main article due to space constraints. A maximum of ten Extended Data display items (figures and tables) is permitted.

Finally, please ensure that you retain unprocessed data and metadata files after publication, ideally archiving data in perpetuity, as these may be requested during the peer review and production process or after publication if any issues arise.

Please use the link below to submit a revised paper:

Link Redacted

Note: This url links to your confidential homepage and associated information about manuscripts you may have submitted or be reviewing for us. If you wish to forward this e-mail to co-authors, please delete this link to your homepage first.

Nature Microbiology is committed to improving transparency in authorship. As part of our efforts in this direction, we are now requesting that all authors identified as ‘corresponding author’ on published papers create and link their Open Researcher and Contributor Identifier (ORCID) with their account on the Manuscript Tracking System (MTS), prior to acceptance. This applies to primary research papers only. ORCID helps the scientific community achieve unambiguous attribution of all scholarly contributions. You can create and link your ORCID from the home page of the MTS by clicking on ‘Modify my Springer Nature account’. For more information please visit please visit www.springernature.com/orcid.

If you wish to submit a suitably revised manuscript we would hope to receive it within 6 months. If you cannot send it within this time, please let us know. We will be happy to consider your revision, even if a similar study has been accepted for publication at Nature Microbiology or published elsewhere (up to a maximum of 6 months).

Looking forward to the revised version!

Best wishes,

****

Reviewer Expertise:

Referee #1: AhR signalling
Referee #2: Malassezia, Tryptophan metbolism
Referee #3: Malassezia physiology. skin microbiome
Referee #4: Skin microbiome, fungi

Reviewer Comments:

Reviewer #1 (Remarks to the Author):

The manuscript by Gushiken-Ibañez investigates the role of Malassezia furfur in maintaining epidermal integrity. Using a fungal mutant which is not able to produce tryptophan-derived indole metabolites that activate the aryl hydrocarbon receptor (AHR) they show that M. furfur-derived AHR activation restores barrier function and controls inflammation in a keratinocyte intrinsic manner. AHR-deficient mice do not benefit from M. furfur-mediated barrier protection, highlighting the importance of microbial derived AHT activation in skin homeostasis. The novel an important finding presented here build on a growing body of evidence affirming a central role for AHR signalling in skin and provide a new perspective on the functional role of M. furfur, proposing that its metabolic activity is a compensatory response to environmental or host-derived stressors rather than a pathogenic agent in inflammatory disease as atopic dermatitis.
The manuscript is very well written, the experiments are well executed with appropriate controls used throughout, and validation experiments performed where necessary.
Nonetheless, a couple of points deserve the authors’ attention:
1) AHR activation is only measured at 1 time point (24h); how is CYP1A1 expression at earlier time points? This assessment is of importance also given the next point.
2) The observation that M. furfur alone as well as the other strains i elicited a basal AHR activation independently of tryptophan (Fig. 1) is likely due to the presence of Trp in the culture media. The authors should please address /acknowledge this point.

Reviewer #2 (Remarks to the Author):

I have read with extreme interest the manuscript entitled 'The skin commensal yeast Malassezia promotes tissue homeostasis via the aryl hydrocarbon receptor'. The Authors have performed an exhaustive investigation on the interaction of 2 Malassezia furfur species with a variety of skin equivelants (cell cultures, skin equivelants, experimental animals). Their findings significanlty impact our future approach on the interaction of this ubiquitous yeast with the skin and associated skin diseases. Some points that need to be addressed are:
Major points:
There appears to be a selective presentation of statistical analysis in graphs of all figures. There are instances where specific comparisons and their statistical testing is completely ignored.
Also SEM or SD is missing from most figures. Statistical test used should be included in the figure legend, as it is key information, for each subpanel of the figure so the reader doesn't have to go back and forth in the manuscript to find this info.

All figures:

1. Figure 1:

1C. The OD value of the -Trp pigmentation should be added to the plot.
1E label the top most up or down regulated metabolites. Indicate the metabolites of interest on the volcano plot.
Number of samples should be included in Figure legend.
1G-1J: SEM is missing.
All plots should be rederived and show fold change over the control not relative expression as this is difficult to assess and the way it is calculated is not presented in the methods.
Statistics should be repeated based on fold change. Statistical test used should be clearly shown for each subpanel in the corresponding figure legend.
Figure legend for 1G-J is using the wrong letters it is referring to A-D.

2. Figure 2:

2A and 2B change to fold change
Add SEM
Indicate statistical test

2B. AHR DK14 relative expression for Ovol1 and Ivl appears statistically significant based on the data presented. Is it significantly different? if so is this reflecting a baseline effect of the Ahr KD14?

3. Figure 3

3B.Would like to see ear thickness of AHR+/- +M furfur +/- Trp on the same figure panel.

3C. SEM missing. Also include test used.

3C and 3A ear thickness measurements don't agree.
3D % of gated populations should be shown. Otherwise no conclusion can be derived from the shown representative plots.

3F. Stats missing for Ahr -/- day 1. Why does day 1 for Ahr-/- differs from Ahr+/- day 1? Surprisingly the vehicle control has a remarkably similar pattern although the actual measurements appear different.

4. Figure 4

4B. Ahrfl/fl and Ahrk14 should also be included in the same Figure panel.

4C. SEM and test used.

4D day 1 M Furfur +Trp is statistically different ?

5. Figure 5

5A. Change to fold change.
n-=2 for some groups. How were stats performed?

In Results words such as robust, marked etc should be accompanied by the corresponding p values.

Minor concerns:

A small number of typographical errors such as line 194, "possesses a enhanced "

Lines 114-115: 'This effect was mediated....indoles'. The sentence should be rephrased for clarity
Line 127: I am not certain if these two references are appropriate for describing aneuploidy of the strain. Maybe they should be moved after word 'tryptophan'
Line 145: I could not locate the heat inactivations protocol for Malassezia
Line 212-213: Reference 47 might be wrong. Please check.
Line 436-444: The findings of the Authors could be corroborated by the therapeutic use of indirubin in psoriasis (Lin et al Br J Dermatol 2018 Jan;178(1):124-131. doi: 10.1111/bjd.15894. Epub 2017 Dec 14) and the suggestion of the role of Malassezia metabolome in this disease (Gaitanis et al. Br J Dermatol
. 2018 Sep;179(3):800. doi: 10.1111/bjd.16807. Epub 2018 Jul 4.)

Reviewer #3 (Remarks to the Author):

Comments to Authors:
“The skin commensal yeast Malassezia promotes tissue homeostasis via the aryl hydrocarbon receptor.". Gushiken-Ibañez et al Malassezia Indoles Nat 32951

A. What are the noteworthy results?
The authors present a pivotal, novel, and well executed exploration of a mutualistic, host targeted Malassezia/Host interaction. Recent advances in technology have driven a renewed interest in Malassezia and Malassezia/Host interaction, particularly driven by their ubiquitous presence on mammalian skin. This manuscript presents a significant contribution to this literature and the basic biology of human skin.
The authors show that specific Malassezia species produce a wide range of tryptophan dependent metabolites and pigments, the impact of these metabolites on human skin homeostasis and health, and define the specificity by disruption thereof to abrogate the impact. They also demonstrate that Malassezia regulate us, their human host, through specific secretory molecules that drive host gene expression and physiology. This represents a breakthrough in human skin microbiome research with broad implications for human biology and disease therapy.

B. Will the work be of significance to the field and related fields? How does it compare to the established literature? If the work is not original, please provide relevant references.
The work is highly significant in skin microbiome as it is one of the first to show that skin microbes directly regulate human physiology, and in a mutualistic way. The newly discovered potential for Malassezia to play a role in multiple, non-skin diseases, such as pancreatic cancer and Crohn's Disease, expand the implications. While unmentioned by the authors (and which should be), Malassezia also contributes significant morbidity through septic infection, specifically and often M furfur.

C. Does the work support the conclusions and claims, or is additional evidence needed?
The authors present a detailed and well controlled series of experiments which clearly show that secreted Malassezia metabolites play a key role in maintenance of skin homeostasis. However, the specific molecules are not clearly defined and could be of other classes than discussed. It is abundantly clear that the tryptophan-dependent metabolites are actively produced, secreted, and dependent on tryptophan, sulfur, and sul1. However, I did not see where any of the specific molecules proposed were tested to see if they are indeed the actual molecule. This would be useful information, and if it is not one of the listed molecules it in no way detracts from the work to discuss what the specific material may be and next steps to define it. Also, the authors should clarify this throughout the manuscript.
I do not fully appreciate the inclusion of the experiments regarding metabolite production on different carbon and nitrogen sources. This confirms prior work (Mayser et al), and seems a bit out of place. This section could be significantly abbreviated and replaced with important and insufficient discussion of the skin microbial community and the choice of M furfur as a model organism.

D. Are there any flaws in the analysis, interpretation, and conclusions? Do these prohibit publication or require revision?
M furfur is now considered to be rare on human skin. Do any of the more skin relevant species (restricta, arunalokei, globosa, sympodialis) produce indoles? Please describe why M furfur is useful as a model organism.
CBS 1878 is isolated from human skin (seborrheic dermatitis), but 14141 is a septic isolate. Please discuss the implications.
How much tryptophan would be expected on human skin? How does this compare to the amount used in the culture medium? Same for sulfur.
The indole secretion and AHR activation seems limited to M furfur. The authors should be clear throughout that this may be limited to M furfur in vivo. Please add commentary.
What is the relevance of the different nitrogen/carbon source experiments? Which is most relevant to skin?
Were any of the detected metabolites, or a mixture thereof, able to induce AHR signaling? If so, which ones? If not any of those cited, what could be the activator?
It will be important to update the number, apparent biomass, and species of Malassezia found on skin. This will only reenforce the importance.
This is not the first description of a potentially mutualistic effect of Malassezia. This has been proposed in https://doi.org/10.1038/nmicrobiol.2016.106 and several including https://doi.org/10.1016/j.jid.2017.11.034 and https://doi.org/10.1073/pnas.2212533119. This must be corrected, and in general it is not appropriate to claim work to be "the first ever". Remove all instances of this claim.

E. Details:
• Typo in the abstract line 40, should be "remains"
• Overall, the manuscript could use a detailed editorial review to correct the English usage. This would substantially shorten the manuscript and improve the readability.
• Line 88: I do not believe that M furfur is among the most abundant Malassezia on human skin. The authors should consider a more recent skin microbiome review. Note that now M arunalokei is considered among the most abundant.
• Line 93: Substantial evidence implicates Malassezia as causative in seborrheic dermatitis and antifungal therapy is an accepted and effective treatment. Correct and appropriately reference this paragraph through line 101.
• Line 315: correct typo in "transformatns"
• There are numerous typos throughout, such as line 344
• You did not mention the source of all of the fungal strains.
• Define the abbreviation HPK on first usage.
• The methods section on mutant generation could be abbreviated by referencing prior work.

Reviewer #4 (Remarks to the Author):

In this manuscript by Gushiken-Ibañez et al., the investigators have explored the role of the aryl hydrocarbon receptor (AHR) in

sensing Malassezia furfur in human (in vitro) and mouse (in vitro and in vivo) settings. The investigators find that M. furfur metabolizes tryptophan into a variety of metabolites that stimulate keratinocytes via the AHR and that this helps promote epidermal health and healing. These findings are curious in that they contrast with the general idea that M. furfur can be pathogenic by observing that, under the right circumstances, the organism might be supportive. Previous studies have noted indole-containing tryptophan derived production by Malassezia and activation of AHR in vitro. This study is novel in the context of looking specifically at keratinocytes, employing an in vivo skin inflammation model using AHR knockout animals, and employing a genetic screen to identify at least one M. furfur gene that is important for optimal tryptophan metabolite production. The effect of the AHR knockout in the mouse skin inflammation model is especially compelling. Overall, it is an exciting study. There are, however, a handful of shortcomings as presented.

Main points:
Figure 2 relies on using an air-liquid interface culture of the N/TERT1cell line to establish that the M. furfur tryptophan metabolites upregulate a collection of genes associated with skin barrier function. Were these genes also regulated in the Figure 1 culture of the same cell line (albeit in liquid culture) where CYP1A1 only was assessed? Are both culture approaches necessary or informative? Or is this duplicative? Also, the investigators are stimulating here with crude concoctions. The “soluble tryptophan metabolites” the authors claim to be using (for example line 204) is just the culture supernatant, which certainly contains tryptophan metabolites but also contains much more. The conclusion is likely correct, but it would be enhanced by showing that pure forms of the tryptophan metabolite are sufficient to the effects.

In a related observation, is the “organotypic human epidermal equivalents (HEEs)” really the correct language to present to readers? Even if the “HEE” bit is published language? Isn’t this just air liquid interface culture of the N/TERT1cell line in transwells? This is the same cell line used in Figure 1. The language seems to suggest some kind of primary culture or “organoid” like culture, which this is not.

The conclusion that M. furfur “uniquely” induces AHR is incompletely supported by figure 1 and its supplemental data. Culture conditions were screened and optimized for pigment production by M. furfur, and then the resulting conditions were tested with single strains of other Malassezia spp. Would we get the same outcome, for example, if we had optimized the culture conditions for pigment production by an assortment of M. sympodialis strains? To strongly make this statement, further data are required. More culture conditions? More isolates? A genetic explanation of the difference? Otherwise, the conclusion needs to be couched more carefully as a preliminary indication.

Commonly in microbial knockout experiments, the control is to use of a complemented strain. Is there a technical reason that this is not realistic in the Sul1 mutant? If so, this should be explained to readers, and the alternative approach used (two independent knockouts) should be justified.

Figure 6A&B are the “-Trp” samples different from each other? Does the n need to be larger to even these out? How does the interpretation change if the Sul1 knockout produces a different level of AHR induction even in the absence of Trp? In a related observation, statistics need to be consistently described throughout. Is the cutoff designated as “significant” the same in all figures? Is the lack of a comparison shown always meant to be interpreted as “not significant”? This is especially sticky in figures like 6A where the -Trp samples look to be quite different from each other.

Figure 6D-E is incomplete. The investigator need to provide evaluation of colonization efficiency and comparison of the -/+ Trp cultures as evaluated in Figure 3. What would it mean if the ability of Malassezia to proliferate on skin is elevated in the Sul1 knockout? What would it mean if the effects of Trp are still present in the knockout?

Minor points:
The abstract describes the observation as
Figure 1G-J is reported as measuring AHR activation, but this is really measuring CYP1A induction in a couple of cell lines. This is likely to be a measure of AHR activation, but we can’t rule out that something else is doing this. Receptors like LXRα and PPARα also regulate CYP1A. In the next figure the authors will get to testing this specificity in one of the cell lines with a knockout and in cells from a mouse knockout, so the conclusions here should avoid definitive language and be described as “suggestive of” or “consistent with” the idea of AHR activation until the knockouts are employed.

Growth media should be added to the resource table.

Labeling of 1D would be clearer if the right image labeled “Trp” is labeled “(-)” or such. Trp is in all the cultures, as indicated by the top label.

Line 234 – at the first mention of Ahr/K14 mice, a reference should be provided. The data would further benefit from some sort of internal control for the presence of similar numbers of keratinocytes in the compared samples – perhaps mRNA of a keratinocyte marker?

Is there a reason that fig S3A does not include the Ahr knockout? This would have confirmed that the observed Cyp1a regulation indeed is Ahr dependent.

The discussion states “we uncover a new biochemical pathway in Malassezia that controls the production of indole metabolites.” Does the study really do this? There is an observation that a gene called Sul1 is important and that this suggests a role for a sulfide/sulfate source in permitting tryptophan metabolite production. But this not yet a “new pathway”. What is the pathway by which a sulfur source is relevant?

In figure 2B, why is the comparison between WT and KO not shown? Is there a technical reason why this is not realistic given

that the question being asked is “does the induction of Cyp1a1 by +tryp conditioned media depend on AHR?”

Lines 459-464: “Interestingly, while tryptophan metabolism is essential for indole biosynthesis across multiple fungal species, key enzymes previously implicated in indole production in other fungi, such as the aminotransferase TAM1/ARO8/AroH/ mediating the first step of tryptophan degradation in U. maydis, C. glabrata and Aspergillus fumigatus, respectively, [72-74], appear to be dispensable or redundant with other aminotransferases in M. furfur, despite a biochemical confirmation on M. furfur CBS 1878 [75].” This is a bit garbled. And to my reading, reference 75 does not suggest that MfTam1is dispensable in M. furfur.

****

Version 1:

Reviewer comments:

Reviewer #2

(Remarks to the Author)
All questions asked have been thoroughly answered from the Authors and the quality of the manuscript has singificantly improved.

Reviewer #4

(Remarks to the Author)
This is a very nice paper, and the responses to all comments have been sufficiently addressed.

Reviewer #5

(Remarks to the Author)
The authors have meaningfully addressed the reviewer’s comments, and the result is an improved manuscript. The discovery of the role of Malassezia in maintaining epidermal homeostasis through the production of indole based AHR ligands is a major advancement in the skin and AHR fields.
However, there is a major issue in the mouse genotypes used that was not noted in the previously reviews. The Ahr floxed mice used were strain #6203 from Jackson, this strain was generated using embryonic ES cells from a 129SvJ mouse which carries the Ahd allele, which has low affinity version of the Ahr, this strain carries a number of amino acid differences compared to the high ligand affinity Ahb allele found in C57BL6/J (WT) mice. The low affinity for AHR ligands in strain #6203 is due to a valine versus alanine substitution at amino acid position 375, which results in a 10-fold lower affinity for TCDD and other polycyclic aromatic compounds. Thus, the Ahrfl/fl mice are crossed with Cre mice, which all appear to carry the Ahb allele, would yield a mixed Ahr genetic background. For example, strain #006054 (CMV-Cre) carry the Ahb allele since they were backcrossed against C57BL6/J. While the Ahr-/+ line would be of mixed Ahr allele background. This Ahr allele issue makes data generated in C57BL6/J mice difficult to compare to data generated in the Ahrfl/fl mice. A review of Ahr alleles can be found in PMID: 2823093. Overall, at the minimum this information should appear in a limitations section of this article.

Reviewer #6

(Remarks to the Author)
Regarding the comments of Reviewer #3, all points have been addressed with clarifications or additional experiments to address the comments. Important was the addition of the indole metabolites that restores the functional impact of Sul1(delta) mutants.

Decision Letter:

Our ref: NMICROBIOL-25041175A

10th March 2026

Dear Salomé,

Thank you for submitting your revised manuscript "The skin commensal yeast Malassezia promotes tissue homeostasis via the aryl hydrocarbon receptor." (NMICROBIOL-25041175A). It has now been seen by the original referees and their comments are below. The reviewers find that the paper has improved in revision, and therefore we'll be happy in principle to publish it in Nature Microbiology, pending minor revisions to satisfy the referees' final requests and to comply with our editorial and formatting guidelines.

If the current version of your manuscript is in a PDF format, please email us a copy of the file in an editable format (Microsoft Word or LaTex)-- we can not proceed with PDFs at this stage.

We are now performing detailed checks on your paper and will send you a checklist detailing our editorial and formatting requirements in about a week. Please do not upload the final materials and make any revisions until you receive this additional information from us.

Thank you again for your interest in Nature Microbiology Please do not hesitate to contact me if you have any questions.

Sincerely,

Reviewer #2 (Remarks to the Author):

All questions asked have been thoroughly answered from the Authors and the quality of the manuscript has singificantly improved.

Reviewer #4 (Remarks to the Author):

This is a very nice paper, and the responses to all comments have been sufficiently addressed.

Reviewer #5 (Remarks to the Author):

The authors have meaningfully addressed the reviewer's comments, and the result is an improved manuscript. The discovery of the role of Malassezia in maintaining epidermal homeostasis through the production of indole based AHR ligands is a major advancement in the skin and AHR fields.
However, there is a major issue in the mouse genotypes used that was not noted in the previously reviews. The Ahr floxed mice used were strain #6203 from Jackson, this strain was generated using embryonic ES cells from a 129SvJ mouse which carries the Ahd allele, which has low affinity version of the Ahr, this strain carries a number of amino acid differences compared to the high ligand affinity Ahb allele found in C57BL6/J (WT) mice. The low affinity for AHR ligands in strain #6203 is due to a valine versus alanine substitution at amino acid position 375, which results in a 10-fold lower affinity for TCDD and other polycyclic aromatic compounds. Thus, the Ahrfl/fl mice are crossed with Cre mice, which all appear to carry the Ahb allele, would yield a mixed Ahr genetic background. For example, strain #006054 (CMV-Cre) carry the Ahb allele since they were backcrossed against C57BL6/J. While the Ahr-/+ line would be of mixed Ahr allele background. This Ahr allele issue makes data generated in C57BL6/J mice difficult to compare to data generated in the Ahrfl/fl mice. A review of Ahr alleles can be found in PMID: 2823093. Overall, at the minimum this information should appear in a limitations section of this article.

Reviewer #6 (Remarks to the Author):

Regarding the comments of Reviewer #3, all points have been addressed with clarifications or additional experiments to address the comments. Important was the addition of the indole metabolites that restores the functional impact of Sul1(delta) mutants.

Version 2:

Decision Letter:

11th May 2026

Dear Salomé,

Thanks for making the final changes to the manuscript. I am pleased to accept your Article "Commensal yeast Malassezia produces tryptophane metabolites to promote tissue homeostasis via the aryl hydrocarbon receptor in mice" for publication in Nature Microbiology. Thank you for having chosen to submit your work to us and many congratulations.

Over the next few weeks, your paper will be copyedited to ensure that it conforms to Nature Microbiology style. We look particularly carefully at the titles of all papers to ensure that they are relatively brief and understandable.

Once your paper is typeset, you will receive an email with a link to choose the appropriate publishing options for your paper and our Author Services team will be in touch regarding any additional information that may be required. Once your paper has been scheduled for online publication, the Nature press office will be in touch to confirm the details.

You may wish to make your media relations office aware of your accepted publication, in case they consider it appropriate to organize some internal or external publicity. Once your paper has been scheduled you will receive an email confirming the publication details. This is normally 3-4 working days in advance of publication. If you need additional notice of the date and time

of publication, please let the production team know when you receive the proof of your article to ensure there is sufficient time to coordinate. Further information on our embargo policies can be found here:
https://www.nature.com/authors/policies/embargo.html

After the grant of rights is completed, you will receive a link to your electronic proof via email with a request to make any corrections within 48 hours. If, when you receive your proof, you cannot meet this deadline, please inform us at rjsproduction@springernature.com immediately. You will not receive your proofs until the publishing agreement has been received through our system

Due to the importance of these deadlines, we ask you please us know now whether you will be difficult to contact over the next month. If this is the case, we ask you provide us with the contact information (email, phone and fax) of someone who will be able to check the proofs on your behalf, and who will be available to address any last-minute problems.

Acceptance of your manuscript is conditional on all authors' agreement with our publication policies (see https://www.nature.com/nmicrobiol/editorial-policies). In particular your manuscript must not be published elsewhere.

Authors may need to take specific actions to achieve compliance with funder and institutional open access mandates. If your research is supported by a funder that requires immediate open access (e.g. according to Plan S principles or the NIH public access policy) then you should select the gold OA route, and we will direct you to the compliant route where possible. Because authors warrant under our subscription licensing terms that they haven't committed to licensing any version of their article under a licence inconsistent with the terms of our agreement – including the applicable embargo period – publication under the subscription model isn't suitable for authors whose funders require no embargo.

If you have any questions about our publishing options, costs, Open Access requirements, or our legal forms, please contact ASJournals@springernature.com

An online order form for reprints of your paper is available at https://www.nature.com/reprints/author-reprints.html. All co-authors, authors' institutions and authors' funding agencies can order reprints using the form appropriate to their geographical region.

We welcome the submission of potential cover material (including a short caption of around 40 words) related to your manuscript; suggestions should be sent to Nature Microbiology as electronic files (the image should be 300 dpi at 210 x 297 mm in either TIFF or JPEG format). Please note that such pictures should be selected more for their aesthetic appeal than for their scientific content, and that colour images work better than black and white or grayscale images. Please do not try to design a cover with the Nature Microbiology logo etc., and please do not submit composites of images related to your work. I am sure you will understand that we cannot make any promise as to whether any of your suggestions might be selected for the cover of the journal.

You can now use a single sign-on for all your accounts, view the status of all your manuscript submissions and reviews, access usage statistics for your published articles and download a record of your refereeing activity for the Nature journals.

To assist our authors in disseminating their research to the broader community, our SharedIt initiative provides you with a unique shareable link that will allow anyone (with or without a subscription) to read the published article. Recipients of the link with a subscription will also be able to download and print the PDF.

As soon as your article is published, you will receive an automated email with your shareable link.

With kind regards,

P.S. Click on the following link if you would like to recommend Nature Microbiology to your librarian
http://www.nature.com/subscriptions/recommend.html#forms

** Visit the Springer Nature Editorial and Publishing website at www.springernature.com/editorial-and-publishing-jobs for more information about our career opportunities. If you have any questions please click here.**

Open Access This Peer Review File is licensed under a Creative Commons Attribution 4.0 International License, which permits use, sharing, adaptation, distribution and reproduction in any medium or format, as long as you give appropriate credit to the original author(s) and the source, provide a link to the Creative Commons license, and indicate if changes were made.
In cases where reviewers are anonymous, credit should be given to 'Anonymous Referee' and the source.
The images or other third party material in this Peer Review File are included in the article's Creative Commons license, unless indicated otherwise in a credit line to the material. If material is not included in the article's Creative Commons license and your intended use is not permitted by statutory regulation or exceeds the permitted use, you will need to obtain permission directly from the copyright holder.
To view a copy of this license, visit https://creativecommons.org/licenses/by/4.0/

Reviewer #1 (Remarks to the Author):

The manuscript by Gushiken-Ibañez investigates the role of Malassezia furfur in maintaining epidermal integrity. Using a fungal mutant which is not able to produce tryptophan-derived indole metabolites that activate the aryl hydrocarbon receptor (AHR) they show that M. furfur-derived AHR activation restores barrier function and controls inflammation in a keratinocyte intrinsic manner. AHR-deficient mice do not benefit from M. furfur-mediated barrier protection, highlighting the importance of microbial derived AHT activation in skin homeostasis. The novel an important finding presented here build on a growing body of evidence affirming a central role for AHR signaling in skin and provide a new perspective on the functional role of M. furfur, proposing that its metabolic activity is a compensatory response to environmental or host-derived stressors rather than a pathogenic agent in inflammatory disease as atopic dermatitis. The manuscript is very well written, the experiments are well executed with appropriate controls used throughout, and validation experiments performed where necessary. Nonetheless, a couple of points deserve the authors' attention:

Author reply: We thank the reviewer for the positive feedback on our work, for highlighting the relevance of our findings in light of the physiological role of the abundant skin fungus and for acknowledging the quality of the experimental work and the text.

1) AHR activation is only measured at 1 time point (24h); how is CYP1A1 expression at earlier time points? This assessment is of importance also given the next point.

Author reply: In response to the reviewer's comment, we stimulated HaCaT keratinocytes with *M. furfur*-derived metabolites for 6 hours and observed an induction of *CYP1A1* expression, confirming early AhR activation. These new results are included in the revised manuscript (line 192) and presented in the new Extended Data Figures 1B-C.

2) The observation that M. furfur alone as well as the other strains i elicited a basal AHR activation independently of tryptophan (Fig. 1) is likely due to the presence of Trp in the culture media. The authors should please address /acknowledge this point.

Author reply: We agree with the reviewer that the basal AHR activation observed in response to incubation with *M. furfur* yeast cells grown in absence of Trp over medium is likely due to metabolic activity of the fungus in response to Trp contained in the culture medium. However, when stimulating keratinocytes with conditioned medium, the response to conditioned medium from *M. furfur* grown in absence of tryptophan did not differ significantly from that of untreated keratinocytes (see e.g. Figures 1I-J and Supplementary Figure S2 in the original manuscript). As Reviewer #2 asked us to display all RT-qPCR data as fold change expression over control, the basal AHR activation is no longer apparent, and we have therefore removed all statement linked to the basal response (line 189 - 203).

Reviewer #2 (Remarks to the Author):

I have read with extreme interest the manuscript entitled 'The skin commensal yeast Malassezia promotes tissue homeostasis via the aryl hydrocarbon receptor'. The Authors have performed an exhaustive investigation on the interaction of 2 Malassezia furfur species with a variety of skin

equivalents (cell cultures, skin equivalents, experimental animals). Their findings significantly impact our future approach on the interaction of this ubiquitous yeast with the skin and associated skin diseases. Some points that need to be addressed are:

Author reply: We thank the reviewer for the positive feedback and for highlighting the impact of our findings.

Major points:

There appears to be a selective presentation of statistical analysis in graphs of all figures. There are instances where specific comparisons and their statistical testing is completely ignored. Also SEM or SD is missing from most figures. Statistical test used should be included in the figure legend, as it is key information, for each subpanel of the figure so the reader doesn't have to go back and forth in the manuscript to find this info.

Author reply: We thank the reviewer for highlighting these points. In the revised version of the manuscript, we have carefully re-checked all main and Extended Data figures and ensured that statistical analyses are presented consistently across all panels. We now indicate the exact *p*-values (instead of *, **, etc.), alongside mean ± SEM or mean ± SD, as appropriate. In dot plots, which show individual samples/mice, we only indicate the mean. Furthermore, the statistical tests used are now specified for each panel (or group of linked panels), instead of listing them for all panels at the end of the legend of each figure. This however makes the legends significantly longer.

All figures:

1C. The OD value of the -Trp pigmentation should be added to the plot.

Author reply: While the OD_{600} values (growth rate of the culture) were included, the OD_{400} values (pigmentation) were not included for the -Trp group because they were all negative and/or close to zero. The parameter OD_{400} to measure the pigmentation was experimentally determined to have the wavelength where the absorption between Trp-treated and the non-treated cultures differed most.

1E label the top most up or down regulated metabolites. Indicate the metabolites of interest on the volcano plot. Number of samples should be included in Figure legend.

Author reply: We have labeled the most strongly up-regulated metabolites in the volcano plot that are of interest for this study (revised Figure 1E). We have also specified the number of biological replicates in the corresponding figure legend (line 1242).

1G-1J: All plots should be rederived and show fold change over the control not relative expression as this is difficult to assess and the way it is calculated is not presented in the methods. Statistics should be repeated based on fold change. Statistical test used should be clearly shown for each subpanel in the corresponding figure legend.

Author reply: We thank the reviewer for this suggestion. We have re-derived plots showing RT-qPCR results to present data as fold change over control instead of relative expression (Modified Figures 1G-J, 2A-B, 6A-B, revised Figure 6G-I, Extended Data Figures 1B-C, Extended Data Figures 2A-E), as explained in the methods section (line 882), except for Extended Data Figure 1D-E and Extended Data Figure 3A-B which do not comprise an untreated group. All statistical analyses have been redone for the data now displayed as fold change and the tests used are indicated in the legend to each panel.

1G-1J: SEM is missing.

Author reply: As explained above, in dot plots, which show individual samples/mice, we only indicate the mean.

Figure legend for 1G-J is using the wrong letters it is referring to A-D.

Author reply: We apologize for this oversight. The letters have been corrected (line 1243).

2A and 2B change to fold change, add SEM, and indicate statistical test.

Author reply: This has been adjusted as explained above for Figure panels 1G-J.

2B. AHR DK14 relative expression for Ovol1 and Ivl appears statistically significant based on the data presented. Is it significantly different? if so, is this reflecting a baseline effect of the Ahr KD14?

Author reply: We believe this reflects a different baseline between the two genotypes. Following this reviewer's suggestion for panels 1G-J, we now present RT-qPCR data as fold change over control. Differences between baselines are no longer apparent.

3B. Would like to see ear thickness of AHR+/- +M furfur +/- Trp on the same figure panel.

Author reply: Ear thickness data for *Ahr*+/– mice colonized with *M. furfur* ± Trp have been included in revised Figure 3B.

3C. SEM missing. Also include test used.

Author reply: As explained above, in dot plots, which show individual samples/mice, we only indicate the mean. The information about the statistical test used was moved from the end of the legend to the respective panel.

3C and 3A ear thickness measurements don't agree.

Author reply: Figure 3A and 3C do not show the same parameters. Panel 3A shows total ear thickness measured *in vivo* with a caliper, whereas panel 3C shows epidermal thickness measured on H&E-stained histology sections at the endpoint. As such, the absolute values (in µm units) are not expected to be the same; however, the relative changes across groups are concordant.

3D % of gated populations should be shown. Otherwise, no conclusion can be derived from the shown representative plots.

Author reply: This has been added, as suggested (revised Figure 3D)

3F. Stats missing for Ahr -/- day 1. Why does day 1 for Ahr-/- differs from Ahr+/- day 1? Surprisingly the vehicle control has a remarkably similar pattern although the actual measurements appear different.

Author reply: We have added the *p*-values for comparison between the *M. furfur* +Trp and *M. furfur* -Trp groups at all time points, including day 1 in revised Figure 3F.

We believe that in the second part of the comment the reviewer is asking why vehicle treated groups are comparable between genotypes, while the *M. furfur*-associated groups are not. Tape stripping impairs skin barrier integrity, which results in an increase in TEWL between d0 and d1. In vehicle-treated groups, this basal increase in TEWL was rapidly restored in an AhR-independent manner. Associating tape-stripped skin with *Malassezia* resulted in much stronger barrier defect than either tape-stripping or fungus alone, as previously reported (PMID: 30870621), and *M. furfur* grown in presence of Trp supports the restoration of the impaired barrier in an AhR-dependent manner. In AhR-deficient skin, the fungus-induced peak of TEWL (day 1) is more pronounced than in AhR-sufficient skin due to the complete defect of barrier restoration by AhR agonists (including *M. furfur*-independent AhR agonists). We added a statement regarding the AhR-independence of the basal increase in TEWL, which was not discussed in the original version of the manuscript (line 298).

4B. Ahrfl/fl and Ahrk14 should also be included in the same Figure panel.

Author reply: The requested conditions have been included in modified Figure 4B.

4C. SEM and test used.

Author reply: *p*-values have been added. As stated above, for dot plots we show individual samples/mice, and therefore no SD/SEM. The information about the statistical test used was moved from the end of the legend to the respective panel.

4D day 1 M Furfur +Trp is statistically different?

Author reply: The *p*-values have been added for all time points.

5A. Change to fold change. n-=2 for some groups. How were stats performed?

Author reply: We believe the reviewer refers to Figure 6A. The number of replicates has been increased, and the display of the data has been changed to fold change relative to -Trp, as suggested by this reviewer for other RT-qPCR datasets (see above). The information about the statistical test used was moved from the end of the legend to the respective panel.

In Results words such as robust, marked etc should be accompanied by the corresponding p values.

Author reply: We adjusted the text (e.g. line 150) and show *p*-values in the accompanying figures wherever we use terminology such as robust, marked etc.

Minor concerns:

A small number of typographical errors such as line 194, "possesses a enhanced "

Author reply: We checked the text throughout for typos.

Lines 114-115: 'This effect was mediated....indoles'. The sentence should be rephrased for clarity.

Author reply: We revised the sentence for more clarity (lines 127)

Line 127: I am not certain if these two references are appropriate for describing aneuploidy of the strain. Maybe they should be moved after word 'tryptophan'

Author reply: We thank the reviewer for spotting this error, which we corrected (lines 143).

Line 145: I could not locate the heat inactivations protocol for Malassezia

Author reply: This has been added to the methods section (line 616).

Line 212-213: Reference 47 might be wrong. Please check.

Author reply: The erroneous reference has been corrected (line 239).

Line 436-444: The findings of the Authors could be corroborated by the therapeutic use of indirubin in psoriasis (Lin et al Br J Dermatol 2018 Jan;178(1):124-131. doi: 10.1111/bjd.15894. Epub 2017 Dec 14) and the suggestion of the role of Malassezia metabolome in this disease (Gaitanis et al. Br J Dermatol. 2018 Sep;179(3):800. doi: 10.1111/bjd.16807. Epub 2018 Jul 4.)

Author reply: We thank the reviewer for this suggestion to strengthens the clinical context of our discussion. The suggested references have been incorporated (line 551).

Reviewer #3 (Remarks to the Author):

A. What are the noteworthy results?

The authors present a pivotal, novel, and well executed exploration of a mutualistic, host targeted Malassezia/Host interaction. Recent advances in technology have driven a renewed interest in Malassezia and Malassezia/Host interaction, particularly driven by their ubiquitous presence on mammalian skin. This manuscript presents a significant contribution to this literature and the basic biology of human skin.

The authors show that specific Malassezia species produce a wide range of tryptophan dependent metabolites and pigments, the impact of these metabolites on human skin homeostasis and health, and define the specificity by disruption thereof to abrogate the impact. They also demonstrate that Malassezia regulate us, their human host, through specific secretory molecules that drive host gene expression and physiology. This represents a breakthrough in human skin microbiome research with broad implications for human biology and disease therapy.

Author reply: We thank the reviewer for highlighting the novelty of our work and its importance by stating that "it represents a breakthrough in human skin microbiome research".

B. Will the work be of significance to the field and related fields? How does it compare to the established literature? If the work is not original, please provide relevant references.

The work is highly significant in skin microbiome as it is one of the first to show that skin microbes directly regulate human physiology, and in a mutualistic way. The newly discovered potential for Malassezia to play a role in multiple, non-skin diseases, such as pancreatic cancer and Crohn's Disease, expand the implications. While unmentioned by the authors (and which should be), Malassezia also contributes significant morbidity through septic infection, specifically and often M furfur.

Author reply: We thank the reviewer for emphasizing that this study "is one of the first to show that skin microbes directly regulate human physiology". In response to the reviewer's suggestion, we now mention extracutaneous diseases associated with *Malassezia* as well septic infections (lines 109, 555).

C. Does the work support the conclusions and claims, or is additional evidence needed?

The authors present a detailed and well controlled series of experiments which clearly show that secreted Malassezia metabolites play a key role in maintenance of skin homeostasis. However, the specific molecules are not clearly defined and could be of other classes than discussed. It is abundantly clear that the tryptophan-dependent metabolites are actively produced, secreted, and dependent on tryptophan, sulfur, and sul1. However, I did not see where any of the specific molecules proposed were tested to see if they are indeed the actual molecule. This would be useful information, and if it is not one of the listed molecules it in no way detracts from the work

to discuss what the specific material may be and next steps to define it. Also, the authors should clarify this throughout the manuscript.

Author reply: Thank you for raising this important point. We have conducted new experiments to directly test candidate indoles for their capacity to rescue the activity of the *sul1* mutant. For this, we first supplemented *sul1*Δ-derived conditioned medium (deficient in several tryptophan-derived indoles, Figure 5K) with indole-3-lactic acid (ILA) and indole-3-acetic acid (IAA)—two commercially available metabolites detected in the metabolome of WT +Trp but absent in the metabolome of *sul1*Δ +Trp, which restored AhR signaling and barrier-gene induction in epidermal sheets. We also conducted *in vivo* rescue experiments with ILA and IAA: Topical administration of the two indoles was sufficient to restore barrier function in two complementary settings: (i) skin colonized with WT *M. furfur* grown in absence of Trp and (ii) skin colonized with *sul1*Δ grown in presence of Trp. In both cases, ΔTEWL and ear thickness were reduced in indole treated compared to solvent-treated control groups. These results are shown in new Figure panels 6G-N. The text was adjusted to incorporate the new data (results line 438, discussion: 505, 509, methods: 664). While these new data show clearly that ILA and IAA, two Sul1-dependent fungal indoles, are sufficient to drive the keratinocyte program linked to barrier restoration, we also added a statement that additional indoles beyond ILA and IAA may also be involved (lines 511).

I do not fully appreciate the inclusion of the experiments regarding metabolite production on different carbon and nitrogen sources. This confirms prior work (Mayser et al), and seems a bit out of place. This section could be significantly abbreviated and replaced with important and insufficient discussion of the skin microbial community and the choice of M furfur as a model organism.

Author reply: We did not test metabolite production by M. furfur in the presence of different carbon and nitrogen sources, but, as clearly stated in the text (lines 151-165), the experiments of Figure 1D aimed to determine whether tryptophan is used as a nitrogen or carbon source to achieve the highest pigmentation. Moreover, the conditions developed in Figure 1D were pivotal for the experiments of figure 5 with the sul1 mutant, as the use of a minimal medium allowed to test sulphate dependency and how this impacted the ability of indole production by the sul1 mutant (Figure 5 G – I). Without these experiments we would not have been able to understand why the sul1 mutant is not producing pigments. Mayser et al used a different medium that included only Tween 80 as a nutrient source, but unfortunately it did not support growth and pigmentation of our parental strain CBS14141 (extended data figure 1).

D. Are there any flaws in the analysis, interpretation, and conclusions? Do these prohibit publication or require revision?

M furfur is now considered to be rare on human skin. Do any of the more skin relevant species (restricta, arunalokei, globosa, sympodialis) produce indoles? Please describe why M furfur is useful as a model organism.

Author reply: Thank you for raising this point. We don't consider *M. furfur* a model organism, but we focused on it for several reasons: (i) *M. furfur* exhibits the strongest AhR activation when compared to the other species tested (extended data figure 1F, G). (ii) *M. furfur* is increased in relative abundance in AD, SD and PV lesional skin (doi: 10.1111/jdv.18347; doi:

10.1038/jid.2013.92; 10.3389/fcimb.2020.00112), suggesting that it has a role in the disease context. As such, we postulate that the indole/AhR-dependent host protective effect of *M. furfur* serves as a mechanism by which the fungus antagonizes barrier disruption and inflammation and to restore homeostasis. (iii) the haploid *M. furfur* strain CBS14141 has a high-quality genome annotation and is tractable for genetic manipulation, which enabled us to provide insight into the genetics of the AhR agonist production. We do not exclude a contribution from other *Malassezia* species, which can also activate AhR, as we show in Extended Data Figure 1F, G, albeit to lower degree under our experimental conditions.

We edited the introduction with respect to our hypothesis of why *M. furfur* may be a particular strong indole producer and activator of the AhR signaling in (barrier-disrupted) skin (line 106)

We also edited the statement about species prevalence (line 89). We don't find any published evidence that *M. arunalokei* is among the most abundant species on the skin - perhaps this is a confidential information that the referee has. Nevertheless, we now mention *M. arunalokei* in the manuscript (line 91).

CBS 1878 is isolated from human skin (seborrheic dermatitis), but 14141 is a septic isolate. Please discuss the implications.

Author reply: The fact that CBS14141 is a septic isolate does not mean that this strain is pathogenic and CBS1878 is not. Microbial pathogenesis is determined to a large extent by the host status and context (e.g., barrier injury, immune status, metabolic environment, etc.), and as such, the site-of-isolation alone is a poor predictor of the pathogenicity potential of a strain, (PMID: 28176789). Importantly, CBS14141 and CBS1878 have been passaged under laboratory conditions (e.g. in rich mDixon medium) for many years, which may have impacted the strains' original property. For example, when exposed to reconstructed human epidermis, the two strains exhibit similar capacity to engage with the host interface (PMID: 39182564).

How much tryptophan would be expected on human skin? How does this compare to the amount used in the culture medium? Same for sulfur.

Author reply: Tryptophan is present in the stratum corneum of the skin as part of natural moisturizing factor (PMID 20394632). Sweat also contains tryptophan (PMID 34045580), providing a low but persistent input over time. The availability of sulfur on the skin surface is limited yet continuously renewed via epidermal turnover and sweat, which harbors sulfur-pathway metabolites (e.g., cysteine/methionine pathway components, PMID 32222072). In our experimental studies we deliberately pre-treated *Malassezia* with above-physiological concentrations of tryptophan to elicit indole production prior to skin colonization, rather than chronically exposing skin to tryptophan, to mechanistically dissect the functional consequences of indole production by the fungus. We now discuss this in line 514).

The indole secretion and AHR activation seems limited to M furfur. The authors should be clear throughout that this may be limited to M furfur in vivo. Please add commentary.

Author reply: In our comparative analysis, diverse *Malassezia* spp. (*M. restricta, M. globosa, M. sympodialis, M. pachydermatis*) were found to activate AhR, albeit to a lower extent compared to *M. furfur* under identical tryptophan-supplemented conditions (Extended Data Figure 1E). The physiological contribution of the different species *in vivo* remains unresolved and may likely also depends on anatomical niche, host factors, and nutrient availability (tryptophan and sulfur-pathway metabolites). We focused our study on *M. furfur* because of its strong activity, and genetic tractability. We have revised the text to emphasize that our findings refer to *M. furfur* and that the physiological contribution of other species to AhR activation remain currently unknown (lines 464).

What is the relevance of the different nitrogen/carbon source experiments? Which is most relevant to skin?

Author reply: Our experimental conditions were designed to mechanistically dissect whether tryptophan serves as a carbon and/or nitrogen source and to investigate how carbon and/or nitrogen availability allow or constrain indole/pigment biosynthesis, rather than to accurately recreate the conditions in the skin niche. Our results indicate that indole production is maximal when tryptophan is available as a nitrogen source together with an additional carbon source and that it is reduced under ammonium-replete conditions (Fig. 1D). Our experimental design further allowed us to establish the sulfate dependence of Sul1-mediated indole production and how this influence the indole production by the WT (Figure 5H–I; Extended Data Figure 5A).

Were any of the detected metabolites, or a mixture thereof, able to induce AHR signaling? If so, which ones? If not any of those cited, what could be the activator? It will be important to update the number, apparent biomass, and species of Malassezia found on skin. This will only reenforce the importance.

Author reply: In response to this reviewer's point C above we tested the activity of ILA and IAA and showed that these two *Malassezia*-derived indoles, whose secretion is abolished in *sul1Δ M. furfur*, are sufficient to activate AhR signaling and to restore the keratinocyte barrier program in murine epidermis when added to *sul1Δ* mutant-derived conditioned medium, recapitulating the effect of *M. furfur* WT +Trp metabolites. In vivo, topical treatment with ILA and IAA restricted *M. furfur*-exacerbated ΔTEWL and ear thickness in two settings—WT *M. furfur* −Trp and *sul1Δ M. furfur* +Trp. These new data are shown in the new Figure panels 6G-N and discussed in the text in lines 438 (results), 505, 509 (discussion), 664 (methods).

In response to the second part of the reviewer's question, we updated the number of currently known *Malassezia* species and revised their role in disease (lines 89)

This is not the first description of a potentially mutualistic effect of Malassezia. This has been proposed in https://doi.org/10.1038/nmicrobiol.2016.106 and several including https://doi.org/10.1016/j.jid.2017.11.034 and https://doi.org/10.1073/pnas.2212533119. This must be corrected, and in general it is not appropriate to claim work to be "the first ever". Remove all instances of this claim.

Author reply: We have removed "first demonstration"/"first ever" language throughout the manuscript. While it has been proposed previously that *Malassezia* can exert mutualistic effects, which we now discuss (line 110), these examples refer to indirect mechanisms by which the fungus benefits the host. As such, it has been shown that *Malassezia* can antagonize *S. aureus* biofilm formation (*in vitro*, PMID 29246799) and skin colonization (in mice, PMID: 40233753). In contrast, our study reveals a direct mechanism, by which *Malassezia* promotes skin health, and it uncovers the implicated fungal and host molecular pathways.

E. Details:

• Typo in the abstract line 40, should be "remains"

Author reply: The typo has been corrected.

• Overall, the manuscript could use a detailed editorial review to correct the English usage. This would substantially shorten the manuscript and improve the readability.

Author reply: We would like to highlight that one of the other reviewers stated that "the manuscript is very well written." We will be happy to shorten the manuscript if asked to do so by the editor to meet any word count restrictions.

• Line 88: I do not believe that M furfur is among the most abundant Malassezia on human skin. The authors should consider a more recent skin microbiome review. Note that now M arunalokei is considered among the most abundant.

Author reply: We have revised the text and updated the reference (lines 95).

• Line 93: Substantial evidence implicates Malassezia as causative in seborrheic dermatitis and antifungal therapy is an accepted and effective treatment. Correct and appropriately reference this paragraph through line 101.

Author reply: We agree with the reviewer and have removed the statement. (line 102)

• Line 315: correct typo in "transformatns"

Author reply: The typo has been corrected.

• There are numerous typos throughout, such as line 344

Author reply: We have proof-read the manuscript and corrected the typos.

• You did not mention the source of all of the fungal strains.

Author reply: The missing information was added in the methods section and all fungal strains used in the study are also listed in Supplementary Table S5.

• Define the abbreviation HPK on first usage.

Author reply: This has been done (line 621).

• The methods section on mutant generation could be abbreviated by referencing prior work.

Author reply: as requested, this section has been shortened by citing our previous work and only kept information important for the reader to understand the current work.

Reviewer #4 (Remarks to the Author):

In this manuscript by Gushiken-Ibañez et al., the investigators have explored the role of the aryl hydrocarbon receptor (AHR) in sensing Malassezia furfur in human (in vitro) and mouse (in vitro and in vivo) settings. The investigators find that M. furfur metabolizes tryptophan into a variety of metabolites that stimulate keratinocytes via the AHR and that this helps promote epidermal health and healing. These findings are curious in that they contrast with the general idea that M. furfur can be pathogenic by observing that, under the right circumstances, the organism might be supportive. Previous studies have noted indole-containing tryptophan derived production by Malassezia and activation of AHR in vitro. This study is novel in the context of looking specifically at keratinocytes, employing an in vivo skin inflammation model using AHR knockout animals, and employing a genetic screen to identify at least one M. furfur gene that is important for optimal tryptophan metabolite production. The effect of the AHR knockout in the mouse skin inflammation model is especially compelling. Overall, it is an exciting study. There are, however, a handful of shortcomings as presented.

Author reply: We thank the reviewer for highlighting the novelty of our study and that the in vivo data are compelling. We appreciate the constructive feedback.

Main points:

Figure 2 relies on using an air-liquid interface culture of the N/TERT1cell line to establish that the M. furfur tryptophan metabolites upregulate a collection of genes associated with skin barrier function. Were these genes also regulated in the Figure 1 culture of the same cell line (albeit in liquid culture) where CYP1A1 only was assessed?

Author reply: We did not measure skin barrier genes in medium-submerged N/TERT1 monolayer cultures. Our intent was to monitor proximal AhR engagement (via *CYP1A1* expression) in simple monolayer cultures (Fig. 1), and then to assess skin barrier genes in models that recapitulate epidermal stratification and terminal keratinocyte differentiation—namely air–liquid interface HEEs and ex vivo epidermal sheets (Figure 2; Extended Data Figure 2). We explained this rationale in the original version of the manuscript (see line 224 in the revised manuscript).

Are both culture approaches necessary or informative? Or is this duplicative? Also, the investigators are stimulating here with crude concoctions. The “soluble tryptophan metabolites” the authors claim to be using (for example line 204) is just the culture supernatant, which certainly contains tryptophan metabolites but also contains much more. The conclusion is likely correct, but it would be enhanced by showing that pure forms of the tryptophan metabolite are sufficient to the effects.

Author reply: As stated above, we first used medium-submerged monolayers of keratinocytes to assess proximal AhR signaling. For testing the functional consequences of AhR activation on skin barrier gene expression we used air-liquid interface HEEs and epidermal sheets, which serve complementary purposes: HEEs provide a stratified epithelium composed exclusively of keratinocytes to read out barrier programs, while epidermal sheets from mouse ear skin retain native epidermal architecture and skin-resident cells, including immune cells. Concordant results across these two experimental systems strengthen mechanistic inference and translatability.

To address the complexity of conditioned media, we now include experiments with purified indoles to show that indole-3-lactic acid (ILA) and indole-3-acetic acid (IAA) induce *Cyp1a1* and skin barrier genes (e.g., *Ivl*, *Fgl*) in epidermal sheets. In vivo, topical treatment with ILA and IAA restricted *M. furfur*-exacerbated ΔTEWL and ear thickness in two settings—WT *M. furfur* −Trp and *sul1*Δ *M. furfur* +Trp. These new data show that defined tryptophan-derived indoles that are produced by *M. furfur* in a Trp-dependent manner are sufficient to drive the keratinocyte/AhR program. The new data are shown in the new Figure panels 6G-N and discussed in the text in lines 440 (results), 505, 509 (discussion), 664 (methods).

The text in the results section preceding the experiments with purified indoles was adjusted for more accuracy (e.g. lines 207, 210, 240, 248, 253, 263).

In a related observation, is the “organotypic human epidermal equivalents (HEEs)” really the correct language to present to readers? Even if the “HEE” bit is published language? Isn’t this just air liquid interface culture of the N/TERT1cell line in transwells? This is the same cell line used in Figure 1. The language seems to suggest some kind of primary culture or “organoid” like culture, which this is not.

Author reply: We agree and have replaced “organotypic human epidermal equivalents” with “air–liquid interface cultures of N/TERT1 keratinocytes in transwells” (line 226). We keep the abbreviation “HEE”, which is common in the literature. We would like to point out that the air-liquid interface cultures used in Extended Data Figure 2 are different from the medium-submerged monolayer cultures of keratinocytes used in Figure 1.

The conclusion that M. furfur “uniquely” induces AHR is incompletely supported by figure 1 and its supplemental data. Culture conditions were screened and optimized for pigment production by M. furfur, and then the resulting conditions were tested with single strains of other Malassezia spp. Would we get the same outcome, for example, if we had optimized the culture conditions for pigment production by an assortment of M. sympodialis strains? To strongly make this statement, further data are required. More culture conditions? More isolates? A genetic explanation of the

difference? Otherwise, the conclusion needs to be couched more carefully as a preliminary indication.

Author reply: We have adjusted the text in line 215 to tune down our claim in response to the reviewer's comment. The production of pigments is quite diffuse in many strains of *M. furfur*, and this has been largely reported in the past by Mayser and colleagues. We did not repeat the experiments performed in the past by Mayser et al because it was out of the scope of the present work, we only repeated *M. furfur* strain CBS1878 as a control to compare its pigmentation with strain CBS14141, which was never tested previously. Actually reviewer 3 suggested to delete one important experiment (in figure 1D) because similar info was reported by Mayser, despite the fact that the experiment was carried out in a different manner. If we had included more strains then clearly there was a repetition of experiments done by Mayser. Last, the genetic of this process is largely unknown, and this is likely the first study approaching this biological process using a variety of complementary approaches; a simple genetic/genomics comparison cannot explain the different phenotypes that we observe in the ability of *Malassezia* species to pigment.

Commonly in microbial knockout experiments, the control is to use of a complemented strain. Is there a technical reason that this is not realistic in the Sul1 mutant? If so, this should be explained to readers, and the alternative approach used (two independent knockouts) should be justified.

Author reply: We thank the reviewer for raising this point. The gene *SUL1* has been identified through random mutagenesis, which sometimes lacks of correlation between the observed phenotype and the mutation found. While in *Malassezia* there is no sexual cycle for genetic segregation, complementation of a random mutant is not advisable, first for a technical limitation as in practice only the NAT marker works well, and second for the ectopic integration of the complementing construct. Rather than relying on an insertional mutant with an additional ectopic integration for complementation, we generated two independent, clean *sul1* deletion mutants in the haploid CBS14141, both of which recapitulate the phenotype (i.e. loss of pigmentation/indoles, altered sulfur use, reduced AhR activation – see figure 5 D - I) and the AhR-dependent effects in the host, thereby providing a rigorous alternative to complementation. We would like to add that in non-model organisms it is a common practice to generate a deletion mutant instead of generating a complemented strain. We included this explanation in lines 340-344.

Figure 6A&B are the "-Trp" samples different from each other? Does the n need to be larger to even these out? How does the interpretation change if the Sul1 knockout produces a different level of AHR induction even in the absence of Trp? In a related observation, statistics need to be consistently described throughout. Is the cutoff designated as "significant" the same in all figures? Is the lack of a comparison shown always meant to be interpreted as "not significant"? This is especially sticky in figures like 6A where the -Trp samples look to be quite different from each other.

Author reply: We increased the biological replicates in revised Fig. 6A–B. Data are now shown as fold change of +Trp relative to each strain's −Trp baseline, as suggested by Reviewer #2 for Figure 1G-J§, to show that Trp-dependent AhR induction is Sul1-dependent irrespective of any absolute −Trp differences. Data in Figures 6A and 6B are different with the first using *M. furfur* cells and the second using conditioned medium. Note that differential AhR activation in response to cells vs.

conditioned medium are presented and discussed in Figure 1 for the WT strain. Last, we standardized statistics across figures, showing exact p value for all comparisons and n values.

Figure 6D-E is incomplete. The investigator need to provide evaluation of colonization efficiency and comparison of the -/+ Trp cultures as evaluated in Figure 3. What would it mean if the ability of Malassezia to proliferate on skin is elevated in the Sul1 knockout? What would it mean if the effects of Trp are still present in the knockout?

Author reply: We thank the reviewer for raising this important point. We have included fungal-burden data in a new Figure panel 6F, which shows that the WT and sul1 mutant colonize the skin comparably, hence deletion of Sul1 does not impact fungal skin colonization. The comparison of + - Trp cultures is shown in Figure 5F-G, where we show that sul1Δ has a growth defect in minimal medium but not in mDixon, which is indeed the medium used to produce sul1Δ cells for mouse infection (see material and method lines 662 – 663). The effect of Trp on the sul1Δ mutants has been largely characterized in Figure 5: sul1Δ still produces some indole (Figure 5K), but is not able to efficiently use Trp (it retains Trp in the medium, Figure 5L). However, AhR activation is significantly reduced in response to sul1Δ compared to the parental WT (Figure 6A, B), and is restored by supplementation with only two pure indoles (Figure 6G).

Minor points:

The abstract describes the observation as Figure 1G-J is reported as measuring AHR activation, but this is really measuring CYP1A induction in a couple of cell lines. This is likely to be a measure of AHR activation, but we can't rule out that something else is doing this. Receptors like LXRα and PPARα also regulate CYP1A. In the next figure the authors will get to testing this specificity in one of the cell lines with a knockout and in cells from a mouse knockout, so the conclusions here should avoid definitive language and be described as "suggestive of" or "consistent with" the idea of AHR activation until the knockouts are employed.

Author reply: This is a good point. We have changed the description of the results shown in Fig. 1G–J as "CYP1A1 expression" and phrase it as "consistent with AhR activation" (line 194, 196).

Growth media should be added to the resource table.

Author reply: The description of the growth media included in the "Fungal strains" section,

Labeling of 1D would be clearer if the right image labeled "Trp" is labeled "(-)" or such. Trp is in all the cultures, as indicated by the top label.

Author reply: This has been corrected.

Line 234 – at the first mention of Ahr/K14 mice, a reference should be provided.

Author reply: This has been added.

The data would further benefit from some sort of internal control for the presence of similar numbers of keratinocytes in the compared samples – perhaps mRNA of a keratinocyte marker?

Author reply: We believe that the reviewer is referring to the comparison of *AhR*$^{-/-}$ vs. *AhR*$^{+/-}$ mice or *Ahr*$^{\Delta K14}$ vs. *AhR*$^{fl/fl}$ littermate control mice. We would like to point out that the vehicle groups are comparable between genotypes for all readouts tested, including skin thickness, epidermal thickness and TEWL (Figure 3A, C, F; Figure 4A, C, D), indicating that there are no intrinsic baseline differences between the compared groups of mice.

If the reviewer is referring to the expression of structural proteins by keratinocytes, either monolayer cultures or HEEs, the fold change of expression is based on the relative expression compared to the housekeeping gene, which means that the expression levels has already be normalized to the number of cells (Figure 1G-H, Figure 2, Extended data Figure 2A-D).

Is there a reason that fig S3A does not include the Ahr knockout? This would have confirmed that the observed Cyp1a regulation indeed is Ahr dependent.

Author reply: We agree with the reviewer. No, we do not observe differences in *Cyp1a1* expression within the *Ahr* KO. Data in included in the new Extended Data Figure panel 3B.

The discussion states "we uncover a new biochemical pathway in Malassezia that controls the production of indole metabolites." Does the study really do this? There is an observation that a gene called Sul1 is important and that this suggests a role for a sulfide/sulfate source in permitting tryptophan metabolite production. But this not yet a "new pathway". What is the pathway by which a sulfur source is relevant?

Author reply: We agree and have removed this statement (line 479). As pointed out by the reviewer, in this study we did not really identify the biochemical pathway of indole production. We instead found a role for sulfide/sulfate in permitting tryptophan degradation, probably by serving as cofactor or enzyme responsible for tryptophan deamination.

In figure 2B, why is the comparison between WT and KO not shown? Is there a technical reason why this is not realistic given that the question being asked is "does the induction of Cyp1a1 by +tryp conditioned media depend on AHR?"

Author reply: This has been addressed in the revised Figure 2. All expression datasets are now displayed as fold change (stimulated condition over untreated control) and include the comparison between WT and KO groups. Please also see our response to reviewer #2.

Lines 459-464: "Interestingly, while tryptophan metabolism is essential for indole biosynthesis across multiple fungal species, key enzymes previously implicated in indole production in other fungi, such as the aminotransferase TAM1/ARO8/AroH/ mediating the first step of tryptophan degradation in U. maydis, C. glabrata and Aspergillus fumigatus, respectively, [72-74], appear to be dispensable or redundant with other aminotransferases in M. furfur, despite a biochemical

confirmation on M. furfur CBS 1878 [75].” This is a bit garbled. And to my reading, reference 75 does not suggest that MfTam1is dispensable in M. furfur.

Author reply: We agree with the reviewer and have removed the statement.

Reviewer #5 (Remarks to the Author):

The authors have meaningfully addressed the reviewer's comments, and the result is an improved manuscript. The discovery of the role of Malassezia in maintaining epidermal homeostasis through the production of indole based AHR ligands is a major advancement in the skin and AHR fields.

However, there is a major issue in the mouse genotypes used that was not noted in the previously reviews. The Ahr floxed mice used were strain #6203 from Jackson, this strain was generated using embryonic ES cells from a 129SvJ mouse which carries the Ahd allele, which has low affinity version of the Ahr, this strain carries a number of amino acid differences compared to the high ligand affinity Ahb allele found in C57BL6/J (WT) mice. The low affinity for AHR ligands in strain #6203 is due to a valine versus alanine substitution at amino acid position 375, which results in a 10-fold lower affinity for TCDD and other polycyclic aromatic compounds. Thus, the Ahrfl/fl mice are crossed with Cre mice, which all appear to carry the Ahb allele, would yield a mixed Ahr genetic background. For example, strain #006054 (CMV-Cre) carry the Ahb allele since they were backcrossed against C57BL6/J. While the Ahr-/+ line would be of mixed Ahr allele background. This Ahr allele issue makes data generated in C57BL6/J mice difficult to compare to data generated in the Ahrfl/fl mice. A review of Ahr alleles can be found in PMID: 2823093. Overall, at the minimum this information should appear in a limitations section of this article.

Author reply: We thank the reviewer for pointing out this important aspect regarding the history of the AhR floxed mice. We have added an explanation in the methods section about how AhR knockout animals were backcrossed with C57Bl/6 WT mice and cite the review mentioned by the reviewer. None of the animals used in our study carried a functional low-affinity AhR allele. $Ahr^{\Delta K14}$ mice carried two floxed AhR alleles, as did their $Ahr^{fl/fl}$ littermates. The only difference was the presence or the absence of the K14-cre. Again, none of the animals carried a functional low-affinity AhR allele. Furthermore, we would like to point out that our results were also replicated in C57Bl/6 WT mice treated with an AhR inhibitor.

"Of note, the $AhR^{fl/fl}$ strain was originally generated from 129-derived embryonic stem cells that carry the low-affinity *Ahr* allele (Ahr^{d}) [PMID: 2823093]. Homozygous $AhR^{-/-}$ mice were crossed to WT C57BL/6j, and experiments were conducted with $AhR^{-/-}$ knockouts and $Ahr^{+/-}$ littermates, in which the intact *Ahr* allele is derived from the C57BL/6 background bearing the high-affinity variant of the *AhR* allele (Ahr^{b})."
